# Amlodipine exerts inhibitory effects against glioma stem cells through degrading EGFR and down-regulating its downstream pro-survival pathways

**DOI:** 10.1038/s41420-025-02784-3

**Published:** 2025-10-27

**Authors:** Zengyang Li, Xiaopei Zhang, Ping Wen, Fan Ni, Nanheng Yin, Zhicheng Zhang, Tao Zhong, Feiyu Xia, Jiaxin Pan, liang Liu, Jun Dong

**Affiliations:** 1 Department of Neurosurgery, The Second Affiliated Hospital of Soochow University, Suzhou, China; 2 Department of Neurosurgery, Affiliated Nanjing Brain Hospital, Nanjing Medical University, Nanjing, China

**Keywords:** CNS cancer, Cancer stem cells

## Abstract

Glioblastoma is the most aggressive and lethal primary brain tumor in adults with the poorest prognosis, due to its high therapeutic resistance and rapid recurrence, which is closely associated with glioma stem cells (GSCs), which represent a critical therapeutic target in this refractory malignancy. As a classical calcium channel blocker (CCB), amlodipine exhibits exact anti-tumor effect independent of CCB activity. The present study further investigated its effects on GSCs and elucidated the relevant molecular mechanisms. Our results revealed that amlodipine exerted multifaceted inhibitory effects on GSCs, including reducing cell viability, self-renewal, invasiveness, and stemness, while enhancing apoptosis and suppressing intracranial tumor growth derived from GSCs. In contrast, other dihydropyridine CCBs and calcium chelators did not exhibit comparable anti-GSC effects at equivalent concentrations, suggesting that the anti-GSC activity of amlodipine is independent of calcium channel blockade. Mechanistically, amlodipine demonstrated high binding affinity to EGFR on the plasma membrane of GSCs, triggering its internalization via clathrin-independent lipid raft-mediated endocytosis. This process leaded to the lysosomal degradation of EGFR, resulting in the downregulation of EGFR protein levels and subsequent inhibition of downstream pro-survival signaling pathways. Taken together, our studies suggest that amlodipine suppresses GSCs-initiated tumor development via degrading EGFR and down-regulating its downstream pro-survival pathways, implying that amlodipine has novel potential as a therapeutic agent targeting GSCs in glioblastoma, deserving further investigations.

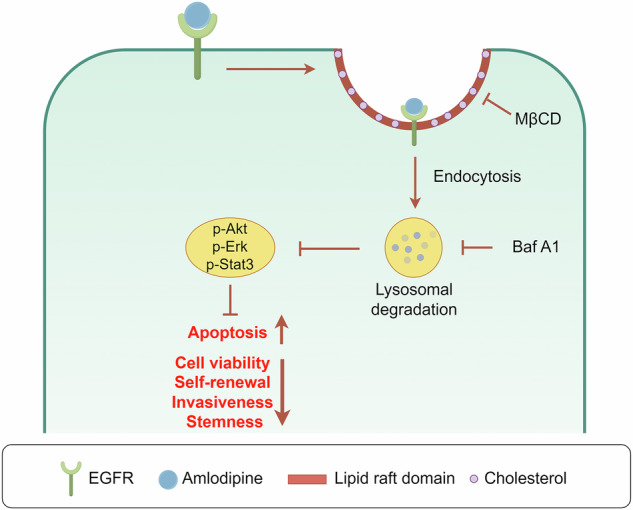

## Introduction

Glioblastoma is the most common and aggressive malignant primary brain tumor in adults, characterized by high therapeutic resistance, rapid recurrence and poor prognosis [1, 2]. The median overall survival of patients is ~15 months [3, 4]. The highly heterogeneous and diffusely infiltrative nature of glioblastoma is largely attributed to glioma stem cells (GSCs), which drive tumor progression through extensive tissue remodeling processes [5, 6].

GSCs possess the hallmarks of self-renewal, multi-lineage differentiation, and high tumorigenic capacity [7–9], which play crucial roles in development of glioblastoma, and represent an important therapeutic target [10, 11]. The development of effective pharmaceutical therapies against GSCs is critical to suppressing GSCs-initiated tumor remodeling and improving patient outcome, however, little progression has been achieved up-to-now [12, 13].

As a calcium channel blocker (CCB), amlodipine has been applied therapeutically against high blood pressure due to its inhibitory effect on calcium ion (Ca^2 +^) entry via interaction with α1 subunit of voltage-dependent L-type Ca^2 +^channel on plasma membrane of vascular smooth muscle cells [14]. Beyond its classical role as a CCB, amlodipine has been reported to exert several pleiotropic effects, including the inhibition of modified low-density lipoprotein (LDL) aggregation, stimulation of nitric oxide (NO) production, antioxidant acting, and smooth-muscle cell proliferation [15, 16]. Furthermore, recent studies have disclosed that amlodipine also exhibits anti-tumor effects on breast and lung cancer by inhibiting cell proliferation, inducing cell cycle arrest and promoting apoptosis, through suppression of the EGFR-Akt/mTOR or Raf/MEK/ERK pathways, instead of CCB activity [17–19].

EGFR (epidermal growth factor receptor) is a receptor tyrosine kinase (RTK) that regulates cell survival, growth, proliferation and differentiation in response to extracellular signals, and aberrant activation of EGFR is frequently observed in glioblastoma [20, 21]. Transduction of neural stem cells (NSCs) or astrocytes with constitutively active EGFR leads to tumorigenic phenotypes of high-grade gliomas [22]. Overexpression and activation of EGFR regulate its downstream signaling pathways to drive development and progression of glioblastoma [23]. Additionally, EGFR promotes self-renewal, proliferation, and stemness maintenance in GSCs, enhancing their therapeutic resistance [24, 25]. Therefore, inhibition of EGFR signaling impairs GSC proliferation and induces apoptosis [26].

The current studies aimed to investigate the biological effects of amlodipine on GSCs and to explore the underlying molecular mechanisms, for the purpose of exploring the potential of repurposing amlodipine as a novel therapeutic approach for targeting GSCs and retarding glioblastoma progression.

## Results

### Amlodipine exhibited precise inhibitory effects on the viability, proliferation, and invasiveness of GSCs

**Fig. 1:**
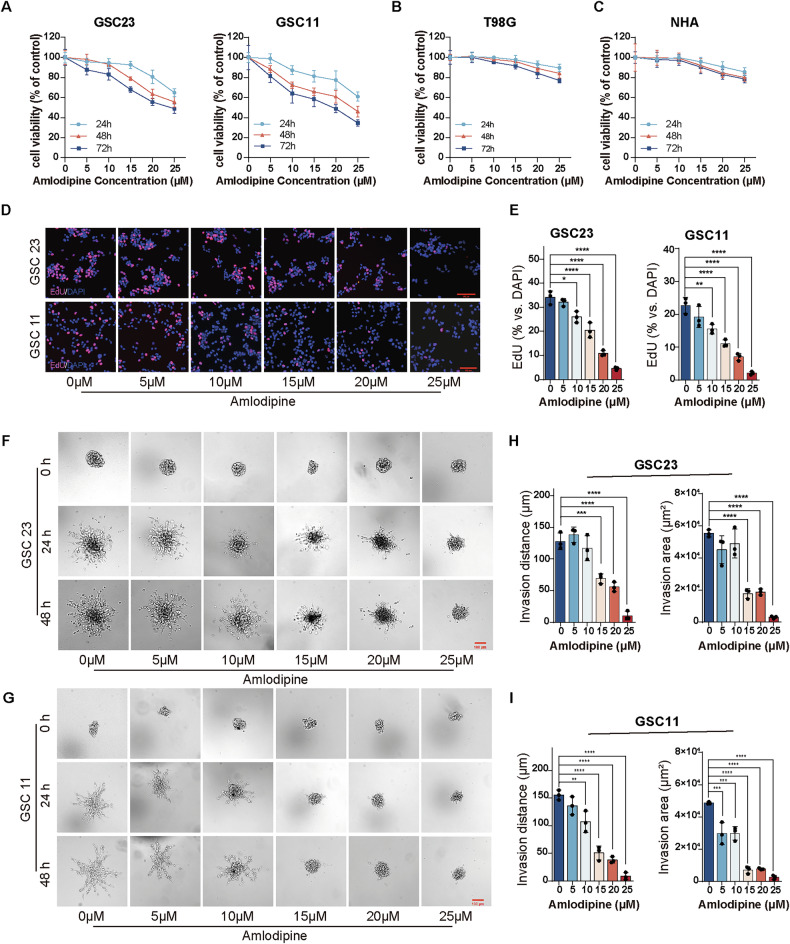
Amlodipine decreased cell viability, suppressed proliferation and invasiveness of GSCs in vitro. **A**–**C** Incubation of GSC23, GSC11, T98G and NHA cells with indicated concentrations of amlodipine (varying from 0 to 25 μM, with 5 μM interval) for 24, 48 and 72 h, respectively. The effect on cell viability of GSC23, GSC11, T98G and NHAs was determined by CCK8 assay. **D** EdU assay detecting cell proliferation (red) of GSC23 and GSC11 cells treated with indicated concentrations of amlodipine (varying from 0 to 25 μM, with 5 μM interval) for 48 h, and counterstained with DAPI (blue) to indicate nuclei, Scale bar = 100 µm. **E** Histogram showing the mean percentage of proliferative cells. **F**, **G** 3D tumor sphere invasion assay to evaluate cell invasion of GSC23 and GSC11 cells treated with indicated concentrations of amlodipine (varying from 0 to 25 μM, with 5 μM interval) for 0, 24, and 48 h, respectively. Scale bar = 100 µm. **H**, **I** The invasion distance and invasion area of GSCs after amlodipine administration were quantitatively analyzed using ImageJ. All data are presented as mean ± SD. *n* = 3 independent experiments. *p < 0.05, **p < 0.01, ***p < 0.001, ****p < 0.0001, compared with the control group.

The CCK8 assay was used to evaluate the effect of amlodipine on GSC23 and GSC11 cells, glioblastoma cells (T98G, SNB19, and LN229), and astrocytes (NHAs). The results showed a significant, dose- and time-dependent decrease in the viability of GSC23 and GSC11 cells (Fig. 1A), whereas glioblastoma cells and NHAs exhibited only a slight, non-statistically significant decline (Fig. 1B, C, Supplementary Fig. S1A). This suggests that amlodipine exerts a strong inhibitory effect primarily on the proliferation of GSCs.

The half maximal inhibitory concentration (IC_50_) value of amlodipine was 34.48 μM for GSC23, and 28.49 μM for GSC11, after administration of amlodipine for 48 h (Supplementary Fig. S1B). Besides, amlodipine significantly hindered EdU incorporation (Fig. 1D), and decreased the percentage of EdU-positive nuclei of GSCs in a dose-dependent manner (Fig. 1E), resulting in a decline in the proliferation of both GSC23 and GSC11.

Amlodipine also significantly impaired the invasive capacity of GSCs, as shown in the 3D spheroid invasion assay (Fig. 1F–G). Both the invasion distance and invasion area were reduced in a dose-dependent manner following amlodipine treatment (Fig. 1H–I), confirming that amlodipine suppresses not only proliferation but also the invasive potential of GSCs.

### Amlodipine induced apoptosis of GSCs and arrested cell cycle of GSCs in G0/G1 phase

**Fig. 2:**
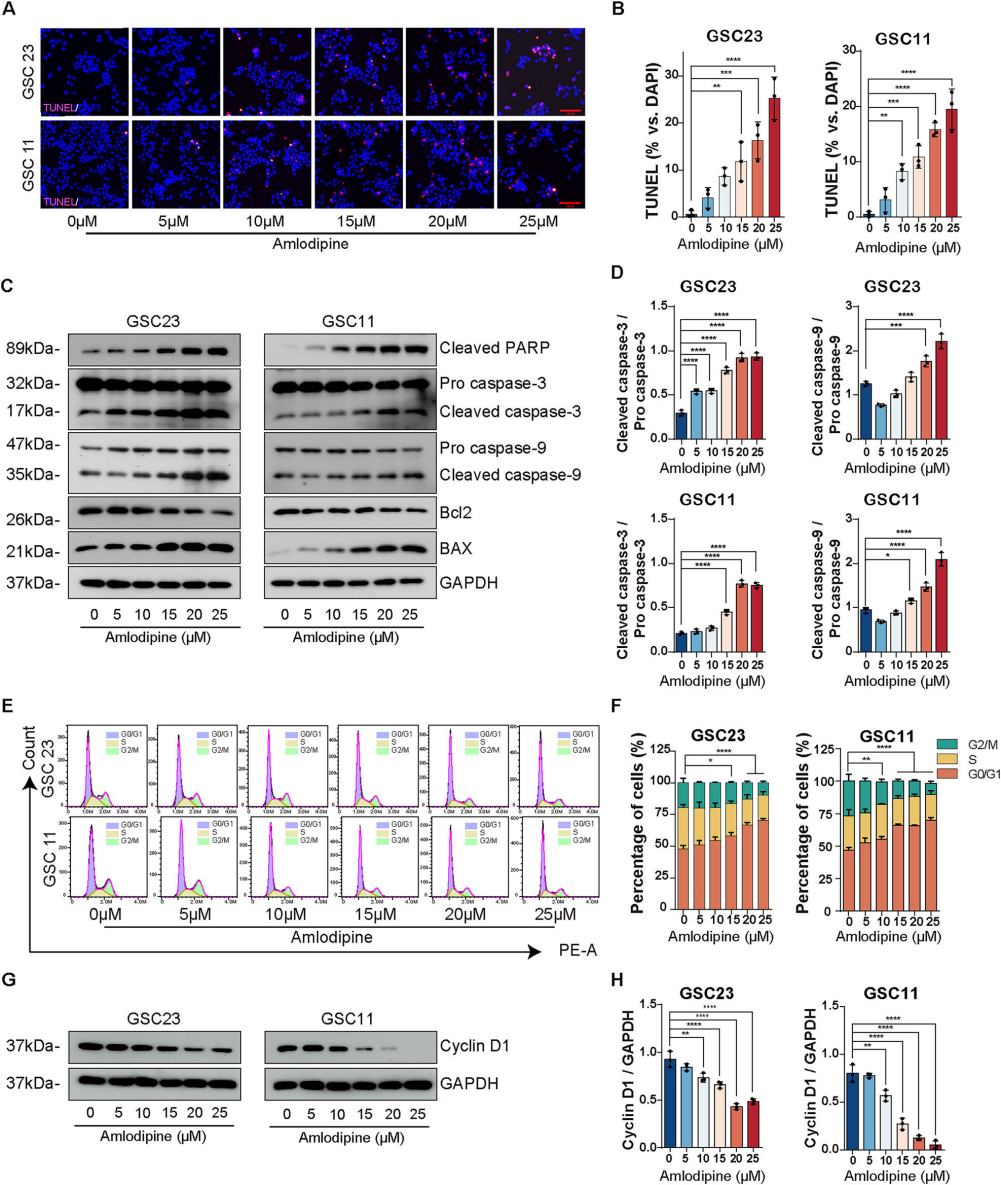
Amlodipine induced apoptosis of GSCs and arrested cell cycle of GSCs in G0/G1 phase. **A** TUNEL assay detecting apoptosis (green) of GSC23 and GSC11 cells treated with indicated concentrations of amlodipine (varying from 0 to 25 μM, with 5 μM interval), and counterstained with DAPI (blue) to indicate nuclei. Scale bar = 100 µm. **B** Elevated percentage of the apoptotic cells was quantified and presented in the corresponding histograms. **C** Western blot detecting protein level of cleaved caspase-3, pro caspase-3, cleaved caspase-9, pro caspase-9, cleaved PARP-1, Bax and Bcl-2 in GSC23 and GSC11 cells after treatment with indicated concentrations of amlodipine (varying from 0 to 25 μM, with 5 μM interval) for 48 h. GAPDH was applied as the loading control. **D** Histograms of relative quantification analysis of the ratio of cleaved caspase-3/pro caspase-3, and cleaved caspase-9/pro caspase-9. **E** PI staining followed with flow cytometry to analyze cell cycle distribution of GSC23 and GSC11 cells after treatment with indicated concentrations of amlodipine (varying from 0 to 25 μM, with 5 μM interval) for 48 h. **F** Histograms showing the percentage of GSC23 and GSC11 cells at G0/G1, S, G2/M phase, respectively. **G** Western blot to detect cell cycle-related protein Cyclin D1 of GSC23 and GSC11 cells after addition of indicated concentrations of amlodipine (varying from 0 to 25 μM, with 5 μM interval) for 48 h. GAPDH was applied as the loading control. **H** Histograms of relative quantification analysis of Cyclin D1/GAPDH ratio. All data are presented as mean ± SD. *n* = 3 independent experiments. *p < 0.05, **p < 0.01, ***p < 0.001, ****p < 0.0001, compared with the control group.

Apoptotic induction in GSCs following amlodipine treatment was evaluated using TUNEL staining. After 48 h of exposure, a significant dose-dependent increase in TUNEL-positive nuclei was observed in both GSC23 and GSC11 cells (Fig. 2A, B). Consistently, Annexin V–FITC/PI dual-staining flow cytometry analysis further confirmed that amlodipine significantly induced apoptosis in a dose-dependent manner (Supplementary Fig. S2A, B).

Western blot analysis revealed that amlodipine upregulated the expression of pro-apoptotic proteins, including cleaved PARP, cleaved caspase-3, cleaved caspase-9, and Bax, while it downregulated the anti-apoptotic protein Bcl-2 in both GSC lines (Fig. 2C, D), suggesting activation of the intrinsic apoptotic pathway.

To assess the effect of amlodipine on cell cycle progression, PI based flow cytometry was performed. Results showed that amlodipine increased the proportion of GSCs in the G0/G1 phase, suggesting moderate G0/G1 cell cycle arrest (Fig. 2E, F).

Further confirmation was provided by Western blotting, which showed a dose-dependent decrease in Cyclin D1 expression, a key regulator of the G1/S phase transition, after 48 h of amlodipine treatment (Fig. 2G, H). These findings collectively indicate that amlodipine induces apoptosis and inhibits cell cycle progression by arresting GSCs in the G0/G1 phase.

### Amlodipine inhibited stemness and self-renewal of GSCs

**Fig. 3:**
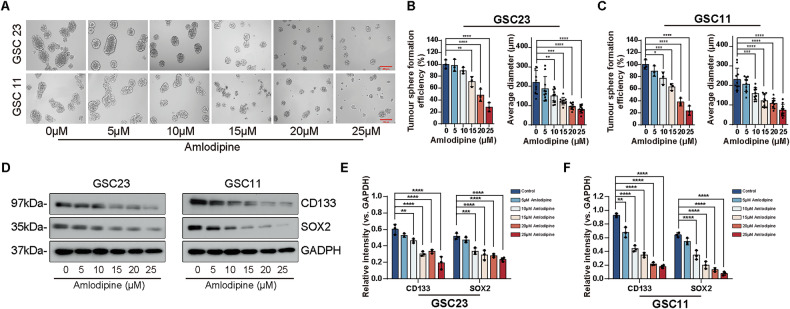
Amlodipine inhibited stemness and self-renewal of GSCs. **A** Representative images of spheroid formation of GSC23 and GSC11 cells after exposure to indicated concentrations of amlodipine (varying from 0 to 25 μM, with 5 μM interval) for 7 days. Scale bar = 250 µm. **B**, **C** Quantitative analysis of sphere formation efficiency and average tumor sphere diameter of GSCs before and after amlodipine treatment. **D**–**F** Western blot analysis and quantitative analysis of the expression level of GSC markers (CD133 and SOX2) in GSC23 and CSC11 cells after treatment with indicated concentrations of amlodipine (varying from 0 to 25 μM, with 5 μM interval) for 48 h. GAPDH was applied as the loading control. All data are presented as mean ± SD. *n* = 3 independent experiments. *p < 0.05, **p < 0.01, ***p < 0.001, ****p < 0.0001, compared with the control group.

The effect of amlodipine on self-renewal of GSCs was observed directly via tumorsphere formation assays in GSC23 and GSC11 cultures. GSC spheroids were more stable and maintained in compact architecture in amlodipine-free medium for 7 days, whereas amlodipine administration diminished both the number and size of tumorspheres (Fig. 3A). The formation efficiency of tumorspheres and average diameter of GSC-spheres decreased significantly (Fig. 3B, C), indicating amlodipine suppresses self-renewal capacity of GSCs.

Besides, both mRNA (Supplementary Fig. S3A, B) and protein level (Fig. 3D–F) of GSC markers (CD133 and SOX2) decreased remarkably in a dose- dependent manner. Additionally, the differentiation marker GFAP showed a marked upregulation at the protein level, as indicated by Western blot analysis (Supplementary Fig. S3C, D). These results suggest that amlodipine effectively suppresses the stem-like properties of GSCs by inhibiting both their molecular and functional hallmarks of stemness.

### Amlodipine altered biological characteristics of GSCs through suppressing EGFR and its downstream pro-survival pathways, independent of CCB activity

**Fig. 4:**
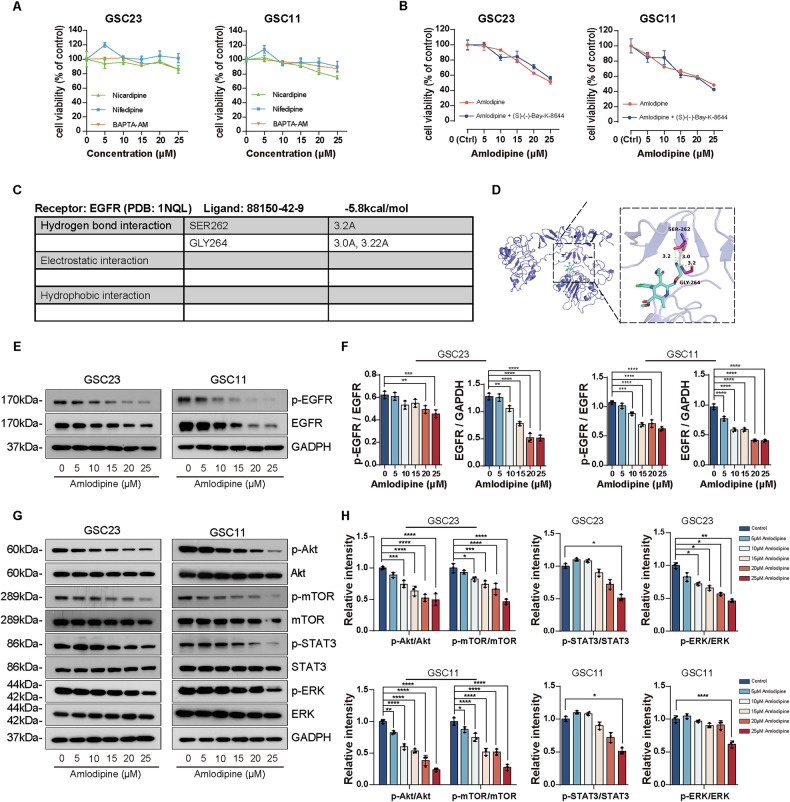
Amlodipine affected the biological characteristics of GSCs through suppressing EGFR and its downstream pro-survival pathways, independent of calcium blocking. **A** Incubation of GSC23 and GSC11 cells with indicated concentrations of nifedipine, nicardipine and Ca^2+^ chelator BAPTA-AM for 48 h, respectively (varying from 0 to 25 μM, with 5 μM interval). The effect of these pharmaceuticals on cell viability of GSC23 and GSC11 cells was determined by CCK8 assay. **B** GSC23 and GSC11 cells were treated with amlodipine (varying from 0 to 25 μM, with 5 μM interval) alone, or combined with (s)-(-)-Bay-K-8644 (200 nM) for 48 h. The effects on cell viability of GSC23 and GSC11 cells was determined by CCK8 assay. **C**, **D** Schematic view of molecular docking of amlodipine and EGFR, protein structure of EGFR was represented as a slate cartoon model, amlodipine was shown as a cyan stick, and their binding sites were shown as magentas stick structures. The hydrogen bond was depicted as yellow dashed lines. **E** Western blot to assay the total and phosphorylated EGFR level of GSC23 and GSC11 cells after addition of indicated concentrations of amlodipine (varying from 0 to 25 μM, with 5 μM interval) for 48 h. **F** Quantitative analysis of total EGFR level, and p-EGFR/EGFR ratio. **G** Western blot to detect expression level of Akt, p-Akt, mTOR, p-mTOR, ERK, p-ERK, STAT3 and p-STAT of GSC23 and GSC11 cells after addition of indicated concentrations of amlodipine (varying from 0 to 25 μM, with 5 μM interval) for 48 h. **H** Quantitative analysis of the ratio of expression level of p-AKT/AKT, p-mTOR/mTOR, p-STAT3/STAT3, and p-ERK/ERK. All data are presented as mean ± SD. *n* = 3 independent experiments. *p < 0.05, **p < 0.01, ***p < 0.001, ****p < 0.0001, compared with the control group.

To determine whether the anti-GSC effects of amlodipine are related to its classical L-type CCB activity, other dihydropyridine CCBs (nifedipine and nicardipine), as well as the intracellular calcium chelator BAPTA-AM, were applied to GSC23 and GSC11 cells. CCK-8 assays showed no obvious change was observed before and after addition of the indicated pharmaceuticals on growth of GSC23 and GSC11cells in vitro (Fig. 4A). Moreover, co-treatment with the L-type Ca^2+^ channel agonist (S)-(–)-Bay-K-8644 (200 nM) failed to reverse the suppressive effect of amlodipine on GSC proliferation (Fig. 4B), further supporting the hypothesis that amlodipine acts via CCB-independent mechanisms.

Previous studies have shown that amlodipine exerts anti-cancer effects by inhibiting EGFR phosphorylation. To further explore this mechanism in GSCs, we first performed molecular docking via Autodock Vina to predict the interaction mode and binding affinity, which disclosed that amlodipine was capable of forming three hydrogen bonds with amino acid residues (SER, GLY) of EGFR, and the binding energy of protein-ligand complex was -5.8 kcal/mol in total, indicating high binding capacity between amlodipine and EGFR (Fig. 4C, D).

Western blot analysis showed that amlodipine significantly decreased total and phosphorylated EGFR expression in both a dose- and time-dependent manner (Fig. 4E, F, Supplementary Fig. S4A, B). The effect of amlodipine on EGFR downstream signaling pathways was evaluated with Western blot, which showed that addition of amlodipine for 48 h reduced the signaling level of p-Akt, p-mTOR, p-STAT3, and p-ERK (Fig. 4G) obviously in dose- and time- dependent manner (Fig. 4G, Supplementary Fig. S4C). Quantitative analysis showed that phosphorylation level of Akt and mTOR reduced most significantly after amlodipine administration, protein level of p-STAT3 and p-ERK decreased moderately (Fig. 4H, Supplementary Fig. S4D).

These results indicate that amlodipine impairs GSC viability and proliferation by suppressing EGFR expression and inhibiting its downstream signaling pathways, particularly the PI3K/Akt/mTOR axis, independent of its classical calcium channel-blocking function.

### Amlodipine enhanced EGFR degradation through activating endolysosomal pathway

**Fig. 5:**
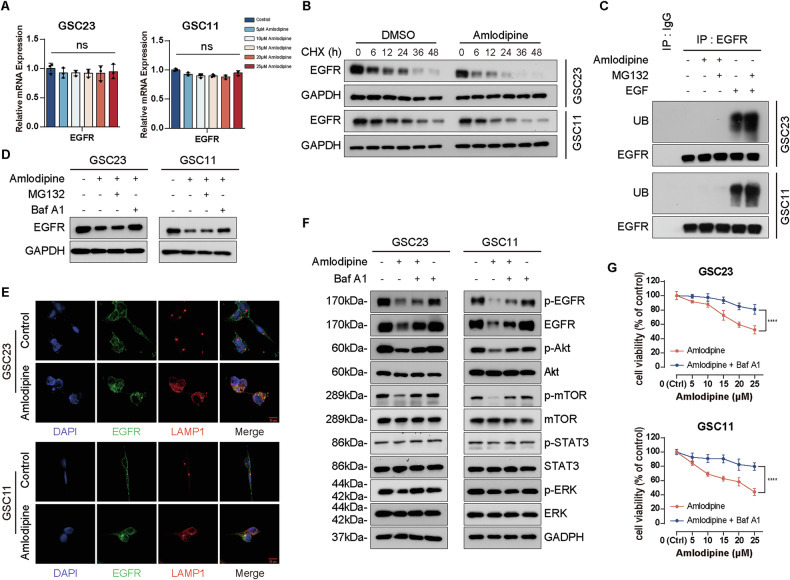
Amlodipine enhanced EGFR degradation through activating lysosomal pathway. **A** After GSC23 and GSC11 cells were treated with amlodipine (varying from 0 to 25 μM, with 5 μM interval) for 48 h, mRNA level of EGFR was analyzed with qRT-PCR. **B** Western blot to detect the protein level of EGFR after GSC23 and GSC11 cells were treated with CHX (20 μM) alone or combined with amlodipine (15 μM) for the indicated times (0, 6, 12, 24, 36 and 48 h). **C** GSC23 and GSC11 cells were treated with amlodipine (15 μM), amlodipine(15 μM) plus MG132(10 μM), EGF (100 ng/ml), or EGF (100 ng/ml) plus MG132(10 μM), respectively for 12 h, then ubiquitination of EGFR was analyzed with Western blot. **D** GSC23 and GSC11 cells were treated with amlodipine (15 μM) alone, or combined either with MG132 (10 μM) or Baf A1 (200 nM), respectively for 48 h, then the protein level of EGFR was assayed with Western blot. **E** GSC23 and GSC11 cells were treated with amlodipine(15 μM) for 48 h, colocalization of EGFR (green) with LAMP1 (red) was detected by confocal immunofluorescence analysis, scale bar = 10 μm. **F** GSC23 and GSC11 cells were treated with amlodipine (15 μM) alone, or combined with Baf A1 (200 nM), respectively for 48 h, then the expression level of total EGFR, p-EGFR, Akt, p-Akt, mTOR, p-mTOR, ERK, p-ERK, STAT3 and p-STAT3 was evaluated with Western blot. **G** GSC23 and GSC11 cells were treated with indicated concentrations of amlodipine (varying from 0 to 25 μM, with 5 μM interval) alone, or combined with Baf A1 (200 nM), respectively for 48 h, then cell viability was detected by CCK8 assay. All data are presented as mean ± SD. *n* = 3 independent experiments. *p < 0.05, **p < 0.01, ***p < 0.001, ****p < 0.0001, compared with the control group.

QRT-PCR assay revealed that amlodipine treatment did not affect mRNA level of EGFR in both GSC23 and GSC11 cells (Fig. 5A). Cycloheximide (CHX, a protein synthesis inhibitor) chase assay was conducted to observe the stability of EGFR without new synthesis, and monitor the rate of EGFR degradation. The results showed that amlodipine plus CHX reduced protein level of EGFR significantly in both GSC23 and GSC11 cells, compared to that of CHX alone (Fig. 5B), indicating that amlodipine enhances EGFR degradation rather than inhibiting its synthesis.

EGFR degradation can proceed through proteasomal or lysosomal pathways. To determine which route was involved, a co-immunoprecipitation assay was used to assess EGFR ubiquitination. The results showed that amlodipine did not increase EGFR ubiquitination, and treatment with the proteasome inhibitor MG132 failed to result in the accumulation of ubiquitinated EGFR (Fig. 5C), suggesting that the proteasome pathway is not involved in amlodipine-induced EGFR degradation.

Then Western blot assay was carried out to further clarify whether lysosome was involved in amlodipine-mediated EGFR degradation, which showed that amlodipine-mediated EGFR reduction was reversed moderately by addition of lysosome inhibitor bafilomycin A1 (baf A1), and addition of proteasome inhibitor MG132 had no effect on EGFR level (Fig. 5D). Besides, immunofluorescence colocalization assay was performed to further verify the lysosomal degradation of EGFR, which disclosed that EGFR (green) in cytoplasm of GSCs colocalizing with lysosomal marker LAMP1(red), observed under confocal microscopic view after amlodipine treatment (Fig. 5E).

Importantly, the suppression of EGFR downstream signaling (p-Akt and p-mTOR) by amlodipine was significantly reversed by Baf A1 co-treatment (Fig. 5F). Additionally, Baf A1 restored cell viability in GSC23 and GSC11 cells treated with amlodipine (Fig. 5G), indicating that lysosomal inhibition mitigates the anti-GSC effects of amlodipine.

These data suggest that the lysosomal degradation pathway mediates EGFR degradation after amlodipine treatment.

### Amlodipine promoted EGFR endocytosis via lipid raft

**Fig. 6:**
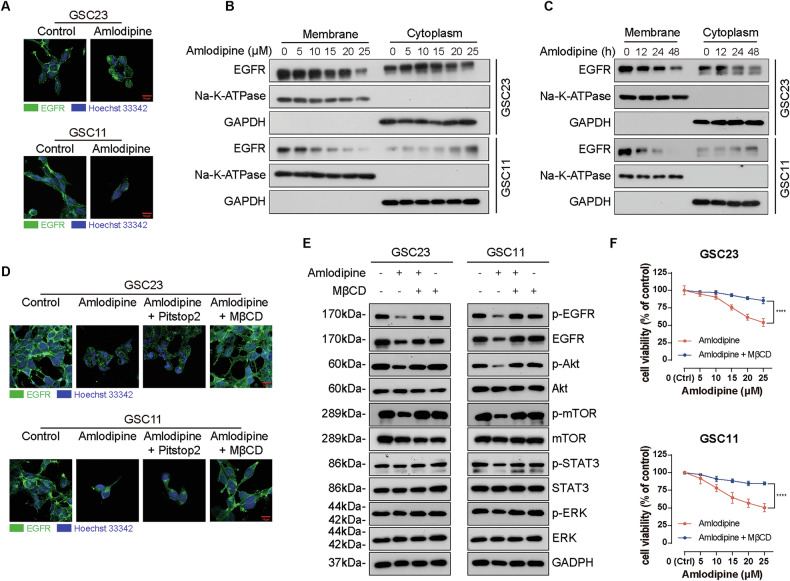
Amlodipine promoted EGFR endocytosis via lipid raft. **A** GSC23 and GSC11 cells were treated with amlodipine(15 μM) for 48 h, then localization of EGFR was detected with confocal immunofluorescent analysis, scale bar = 10 µm. **B** GSC23 and GSC11 cells were treated with indicated concentrations of amlodipine (varying from 0 to 25 μM, with 5 μM interval) for 48 h, then EGFR of cell membrane and cytoplasm was assayed with Western blot. Na-K-ATPase was applied as the loading control as cell membrane protein, GAPDH was applied as the loading control as cytoplasmic protein. **C** GSC23 and GSC11 cells were treated with amlodipine (15 μM) for 12,24 and 48 h, respectively, then EGFR of cell membrane and cytoplasm was detected with Western blot. Na-K-ATPase was applied as the loading control as cell membrane protein, GAPDH was applied as the loading control as cytoplasmic protein. **D** GSC23 and GSC11 cells were treated with amlodipine (15 μM) alone, or combined with either pitstop2 (5 μM) or MβCD (1 mM), respectively for 24 h. EGFR endocytosis was analyzed with confocal immunofluorescent microscopy, scale bar = 10 μm. **E** GSC23 and GSC11 cells were treated with amlodipine (varying from 0 to 25 μM, with 5 μM interval) alone, or combined with MβCD (1 mM), respectively for 48 h, then the protein level of total EGFR, p-EGFR, Akt, p-Akt, mTOR, p-mTOR, ERK, p-ERK, STAT3 and p-STAT3 was assayed with Western blot. **F** GSC23 and GSC11 cells were treated with indicated concentrations of amlodipine (15 μM) alone, or combined with MβCD (1 mM), respectively for 48 h, then cell viability was evaluated with CCK8 assay. All data are presented as mean ± SD. *n* = 3 independent experiments. *p < 0.05, **p < 0.01, ***p < 0.001, ****p < 0.0001, compared with the control group.

To further investigate the mechanism underlying EGFR internalization following amlodipine treatment, IF staining was performed. Amlodipine treatment led to increased cytoplasmic clustering of EGFR, accompanied by a marked reduction of EGFR on the plasma membrane in both GSC23 and GSC11 cells (Fig. 6A). These observations were confirmed by Western blot analysis, which revealed a dose- and time- dependent increase in cytoplasmic EGFR and a corresponding decrease in membrane-associated EGFR after amlodipine exposure (Fig. 6B, C), implying that amlodipine promotes endocytosis of EGFR.

Then IF assay was performed to clarify whether EGFR endocytosis could be mediated by either clathrin or clathrin-independent lipid raft, which disclosed that Pitstop2 (an amphipathic protein-bound inhibitor of clathrin terminal domain) did not suppress amlodipine-induced EGFR endocytosis (Fig. 6D), indicating that amlodipine induced EGFR endocytosis is dependent on clathrin-independent lipid raft.

Given the known role of lipid rafts in clathrin-independent endocytosis, we further tested the involvement of lipid rafts by treating cells with methyl-β-cyclodextrin (MβCD), a cholesterol-depleting agent that disrupts lipid raft structure. MβCD treatment significantly reversed amlodipine-induced EGFR internalization, and restored downstream signaling, as evidenced by elevated levels of p-Akt and p-mTOR (Fig. 6E). Additionally, cell viability was markedly increased in both GSC lines upon MβCD co-treatment (Fig. 6F).

These findings indicate that amlodipine promotes EGFR internalization via a lipid raft–dependent, clathrin-independent pathway. Cholesterol-rich lipid rafts likely serve as an organizational platform for EGFR internalization following interaction with amlodipine, ultimately leading to EGFR degradation and downstream signaling inhibition in GSCs.

### Akt agonist reversed the antitumor effects of amlodipine on GSCs

**Fig. 7:**
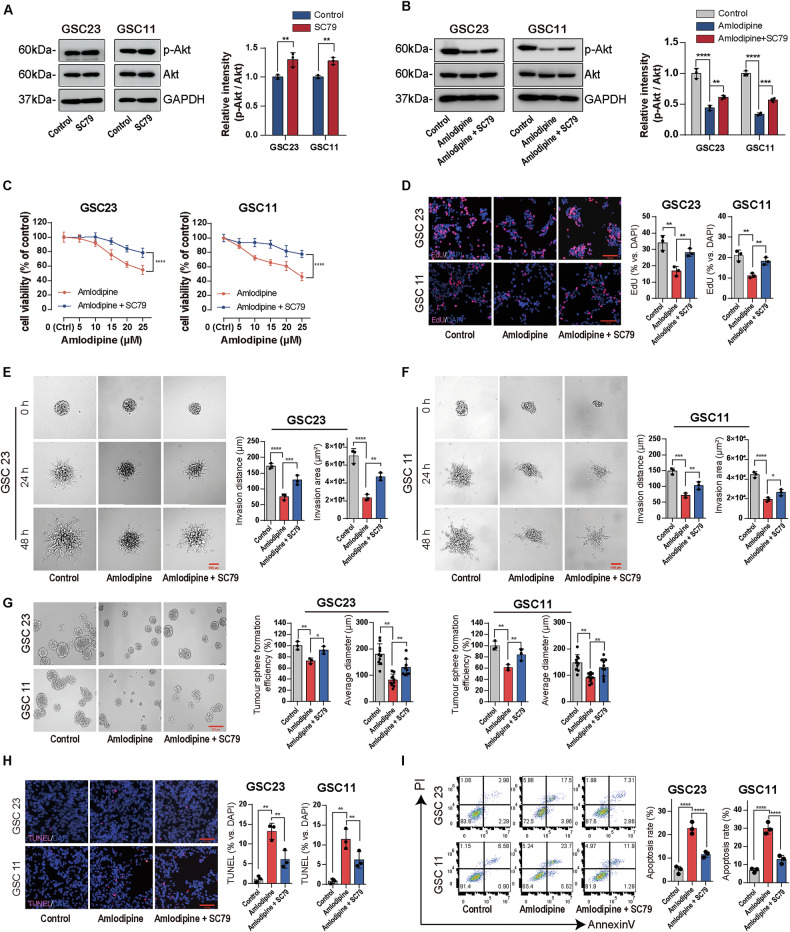
AKT agonist reversed the effects of amlodipine against GSCs. **A** GSC23 and GSC11 cells were treated with SC79 (10 μg/mL) for 30 min, then the protein level of Akt and p-Akt was analyzed with Western blot. **B** GSC23 and GSC11 cells were treated with amlodipine (15 μM) alone, or combined with SC79 (10 μg/mL) for 48 h, then the expression level of Akt and p-Akt was analyzed with Western blot. **C** Incubation of GSC23 and GSC11 cells with amlodipine (varying from 0 to 25 μM, with 5 μM interval) alone, or combined with SC79 (10 μg/mL) for 48 h, the effect on cell viability of GSC23 and GSC11 cells was determined by CCK8 assay. **D** EdU assay detecting cell proliferation (red) of GSC23 and GSC11 cells treated with amlodipine (15 μM) alone, or combined with SC79 (10 μg/mL) for 48 h. Scale bar = 100 µm (left), histograms showing the mean percentage of proliferative cells (right). **E**, **F** 3D tumor sphere invasion assay to evaluate cell invasion capacity of GSC23 and GSC11 cells treated with amlodipine (15 μM) alone, or combined with SC79 (10 μg/mL) for 0, 24 h and 48 h, respectively. Scale bar = 100 µm (left). Quantitative analysis of invasion distance and invasion area of GSCs after indicated treatment (right). **G** Representative images of spheroid formation of GSC23 and GSC11 cells after exposure to amlodipine (15 μM) alone, or combined with SC79 (10 μg/mL) for 7 days. Scale bar = 50 µm(left). Quantitative analysis of sphere formation efficiency and average tumor sphere diameter of GSCs with different treatments (right). **H** Representative images and quantitative analysis of apoptotic GSC23 and GSC11 cells after treatment with amlodipine (15 μM) alone, or combined with SC79 (10 μg/mL) for 48 h, respectively, determined with TUNEL assay. Scale bar = 100 µm. **I** (left) GSC23 and GSC11 cells were harvested after treatment with amlodipine (15 μM) alone, or combined with SC79 (10 μg/mL) for 48 h, respectively, and apoptotic cells were analyzed by Annexin VFITC–PI dual staining flow cytometry. (right) Histograms of mean percentage of apoptotic cells. All data are presented as mean ± SD. *n* = 3 independent experiments. *p < 0.05, **p < 0.01, ***p < 0.001, ****p < 0.0001, compared with the control group.

For further verifying the inhibitory roles of amlodipine against GSCs through downregulating EGFR/Akt/mTOR signaling pathway, GSC23 and GSC11 cells were treated with Akt agonist SC79, a selective Akt agonist. SC79 treatment increased the p-Akt without affecting total Akt protein levels (Fig. 7A).

When SC79 was administered together with amlodipine, a partial restoration of p-Akt expression was observed in both GSC23 and GSC11 cells (Fig. 7B). Functionally, co-treatment with SC79 significantly rescued GSC viability (Fig. 7C), proliferation (Fig. 7D), invasion ability (Fig. 7E, F), and self-renewal capacity (Fig. 7G), all of which had been suppressed by amlodipine alone. Furthermore, the pro-apoptotic effects of amlodipine were also partially attenuated by SC79 co-treatment, as evidenced by a reduction in apoptosis in both cell lines (Fig. 7H–I).

These results demonstrate that activation of Akt by SC79 can partially reverse the antitumor effects of amlodipine, supporting the notion that EGFR/Akt/mTOR pathway inhibition is a key mechanism by which amlodipine suppresses GSC proliferation, invasion, and stemness.

### Amlodipine repressed intracranial growth of GSCs

**Fig. 8:**
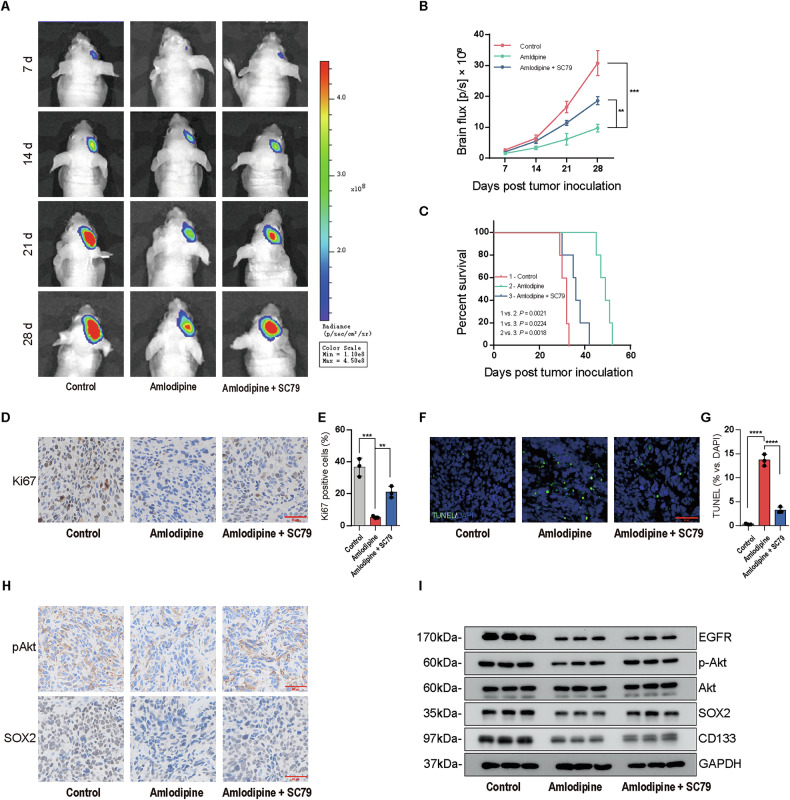
Amlodipine repressed intracranial growth of GSCs-derived xenografts. **A** Representative bioluminescence images of tumor-bearing mice in three groups (amlodipine alone, combined with SC79, and NC group) at 7, 14, 21 and 28 days following implantation of 1 × 10^6^ GSC23-Luc cells. **B** Bioluminescence images were quantitatively analyzed for three experimental groups. Data are means ± SD. *n* = 5 mice per group. ANOVA analysis was applied to evaluate significance. **C** Kaplan−Meier survival curves for three experimental groups. *n* = 5 mice per group. Log-rank analysis was conducted. Control versus amlodipine, *P* = 0.0021; control versus amlodipine plus SC79, *P* = 0.0224; amlodipine versus amlodipine plus SC79, *P* = 0.0018. **D** Representative IHC images of Ki67 expression in tissue section of intracranial GSCs xenografts, Scale bar = 50 µm. **E** Quantitative analysis of Ki67 positive cells in tumor specimen of three experimental groups, ANOVA analysis was applied to evaluate significance. Data are means ± SD. *n* = 3 independent experiments. **F** TUNEL assay of cell apoptosis (green) in GSC23 cells-derived xenografts and counterstained with DAPI (blue) to indicate nuclei. Scale bar = 50 µm. **G** Quantitative analysis of cell apoptosis in GSC23 cells-derived xenografts of three experimental groups. ANOVA analysis was applied to evaluate the significance. Data are means ± SD. *n* = 3 independent experiments. **H** Representative IHC images of expression status of SOX2 and p-Akt (Ser-473) in tumor specimens of three experimental groups, Scale bar = 50 µm. **I** Western blot of expression level of EGFR, p-AKT, AKT, CD133 and SOX2 in GSC23 cells-derived xenografts. *p < 0.05, **p < 0.01, ***p < 0.001, ****p < 0.0001, compared with the control group.

To evaluate the in vivo antitumor effects of amlodipine, an orthotopic intracranial xenograft model using GSC-derived tumors was established. Bioluminescence imaging (BLI) revealed a marked reduction in tumor burden in the amlodipine-treated group compared to the control group (Fig. 8A, B), and overall survival of tumor-bearing mice was significantly prolonged (Fig. 8C). Notably, tumor size was larger and survival shorter in the group receiving amlodipine plus SC79 compared to amlodipine alone, suggesting that activation of Akt partially reverses the therapeutic effects of amlodipine in vivo.

Histopathological analysis showed a significant decrease in Ki67-positive proliferating tumor cells in the amlodipine group compared with the negative control (NC) group. The addition of SC79 attenuated this effect, with a relatively higher proportion of Ki67-positive cells observed (Fig. 8D, E). TUNEL staining of tumor tissues further demonstrated that amlodipine significantly enhanced apoptosis in intracranial tumors compared to the NC group, while co-treatment with SC79 reduced TUNEL positivity, indicating a partial reversal of amlodipine-induced apoptosis (Fig. 8F, G).

In addition, immunohistochemical analysis revealed that the number of SOX2-positive stem-like cells was significantly reduced, and p-Akt expression was clearly suppressed in the amlodipine group (Fig. 8H). These findings were corroborated by Western blot analysis, which showed reduced expression of EGFR, p-Akt, CD133, and SOX2 in amlodipine-treated intracranial xenografts (Fig. 8I), consistent with the in vitro data.

Taken together, these results confirm that amlodipine inhibits the intracranial growth of GSC-derived tumors by reducing proliferation, depleting stemness, and promoting apoptosis. The partial reversal by SC79 further supports that these antitumor effects are mediated through inhibition of the EGFR/Akt/mTOR pathway, suggesting that amlodipine holds significant therapeutic potential against glioblastoma via targeting GSCs.

## Discussion

GSCs exhibit profound therapeutic resistant, driving tumor inevitable recurrence and lethal progression after standardized treatments [27, 28]. Potential therapeutic targets addressing genetic alterations in GSCs include the Wnt/β-catenin, Hedgehog, JAK/STAT, Notch, and PI3K/Akt/mTOR signaling pathways [29–33]. However, these approaches have failed to transform into clinical practice due to low efficacy [34, 35]. Thus, improving the specific therapeutic efficiency against GSCs remains challenging. Drug repurposing, which involves identifying new therapeutic uses for existing clinical drugs, represents an appealing alternative to the costly and lengthy de novo drug development process and holds considerable promise for developing effective anticancer therapies [36].

Previous studies have reported several mechanisms by which amlodipine inhibits cancer progression, including suppression of *PD-L1* expression [37], inhibition of EGFR activity [17, 38], and induction of cell cycle arrest [39] in several cancer types. Additionally, an increasing number of studies have demonstrated that amlodipine exhibits chemo-sensitizing properties against several types of cancer, including leukemia, pancreatic cancer, breast cancer, and lung cancer, primarily by reversing multidrug resistance (MDR) or inducing synergistic apoptosis of cancer cells [40–43]. Furthermore, recent studies have, for the first time, explored the metabolomic changes associated with amlodipine treatment in cancer cells, revealing that amlodipine treatment resulted in significant metabolic alterations in lung cancer cells. The main changes focused on the metabolisms of nicotinate and nicotinamide, arginine and proline, purine, as well as malate-aspartate shuttle pathways. This novel finding highlights the anti-cancer potential of amlodipine via regulating multiple metabolic pathways, further underscoring the multifaceted nature and growing promise of amlodipine as a repurposed anticancer agent [44]. Notably, we have revealed that nicardipine, one of the dihydropyridine CCBs, enhances temozolomide (TMZ) -induced apoptosis in GSCs by repressing autophagy [45]. However, the precise effects of amlodipine on GSCs remained unexplored.

In this study, we demonstrated that amlodipine robustly inhibited GSC viability, proliferation, self-renewal, invasiveness, and stemness, alongside induction of G0/G1 arrest via cyclinD1 upregulation and apoptosis via Bcl-2 downregulation and pro-apoptotic protein upregulation. Critically, other dihydropyridine derivatives, such as nifedipine and nicardipine, did not exhibit consistent inhibitory effects at the same dosage, as well as calcium chelators, though the synergy of nicardipine with temozolomide against GSCs was investigated [45]. Additionally, L-type Ca^2 +^ channel agonist could not counteract the inhibitory effects of amlodipine on GSCs. These findings indicate that inhibitory effects of amlodipine against GSCs are independent of classical CCB activity. While Liu et al. demonstrated that amlodipine activates store-operated calcium entry (SOCE), increasing cytosolic Ca²⁺ to trigger PKCβII-dependent Lats1/2 kinase activation, ultimately inhibiting YAP/TAZ oncogenic signaling via the Hippo pathway [46]. In contrast, our study revealed a distinct mechanism. We discovered that amlodipine binds to EGFR on GSC membranes with high affinity, triggering clathrin-independent lipid raft-mediated endocytosis and subsequent lysosomal degradation, thereby suppressing EGFR/Akt/mTOR signaling. Accumulating evidences have indicated that these two core pathways, which are highly activated and play key roles in progression of glioblastoma, have complex bidirectional interactions. YAP/TAZ, as potent transcriptional co-activators, have been reported to directly or indirectly upregulate the transcription of multiple EGFR ligands, such as Epiregulin and Amphiregulin [47]. These findings suggest that inhibiting YAP/TAZ leads to reduction in EGFR signaling input. Besides, activated EGFR signaling has been shown to negatively regulate the activity of core Hippo pathway kinase Lats1/2 or promote the stability and nuclear translocation of YAP/TAZ [48, 49]. Amlodipine was demonstrated to inhibit both pathways in two independent studies, but further mechanistic studies are needed to precisely elucidate the dynamic relationship between EGFR/AKT and YAP/TAZ under amlodipine exposure.

Based on online molecular docking, high binging affinity of amlodipine with EGFR was predicted in our investigations. Subsequent experimental validation demonstrated significant EGFR phosphorylation inhibition and reduced EGFR protein expression levels following amlodipine treatment in GSCs, both in vitro and in vivo. Protein levels can decrease due to either reduced synthesis or accelerated degradation [50]. Our results confirmed that amlodipine did not affect EGFR synthesis. Further data revealed that amlodipine had no effect on EGFR ubiquitination, lysosome inhibitor (Baf A1), rather than proteasome inhibitor (MG132) could reverse the effect of amlodipine on EGFR degradation. These findings indicated that amlodipine enhanced EGFR degradation not via ubiquitin-proteasomal pathways, but through lysosomal trafficking after internalization.

Endocytosis of membrane-bound EGFR, a prerequisite for lysosomal degradation, occurs via clathrin-mediated (CME) or clathrin-independent (NCE) mechanisms [20, 51]. CME can be inhibited by amphipathic protein-bound inhibitor of clathrin terminal domain (Pitstop2), whereas NCE can be inhibited by MβCD, a cholesterol-depleting agent that disrupts lipid raft structure [52]. Our findings supported the induction of NCE, suggesting the involvement of lipid rafts in this process.

Activated EGFR initiates multiple downstream pro-oncogenic signaling pathways, including PI3K/Akt/mTOR, STAT and Ras/Raf/MEK/ERK pathways. PI3K/Akt signaling pathway is involved in several key cellular functions, including cell growth, survival, apoptosis, motor behaviors and cell metabolism [53]. As a downstream protein of PI3K/Akt, the mammalian target of rapamycin (mTOR) is tightly correlated with tumorigenicity of cancer stem cells (CSCs) [54]. The Ras/Raf/MEK/ERK pathway plays important roles in cell proliferation, differentiation, migration and survival. Abnormal activation of this pathway is closely associated with development of glioblastoma [55]. Recent research showed that ERK1/2 phosphorylation predicts survival following anti-PD-1 immunotherapy in recurrent glioblastoma [56]. The JAK-STAT3 pathway promotes cell proliferation, survival, invasion and metastasis, inhibits apoptosis, as well as plays key roles in maintenance and proliferation of GSCs [57]. We found amlodipine significantly suppressed these downstream pathways, particularly the PI3K/Akt/mTOR cascade. Activation of Akt is critical for neurosphere formation and survival of CD133^+^ GSCs [58]. Our study demonstrated that the Akt activator SC79 partially rescued the suppressive effects of amlodipine on GSC viability, proliferation, invasion, stemness, and apoptosis, further validating the central role of Akt inhibition in amlodipine’s mechanism of action.

Amlodipine can effectively cross the blood-brain barrier [59]. Intraperitoneal administration of amlodipine retarded the intracranial growth of GSCs-derived xenografts and prolonged overall survival of tumor-bearing mice, highlighting the efficacy of amlodipine against GSCs in vivo. IHC and Western blot analyses confirmed decreased expression of EGFR, p-Akt, CD133, and SOX2 in xenograft tissues, aligning well with in vitro. SC79 partially reversed these effects in vivo, further validating the involvement of the EGFR/Akt axis. The dosage of amlodipine applied in the current study was comparable to other studies [17, 60, 61], and no obvious side effect was observed, suggesting its potential for clinical transformation.

Despite recent advances, critical questions still remain. Calcium signaling plays a critical role in regulating the tumor microenvironment, particularly angiogenesis, which is a hallmark of glioblastoma progression and is essential for tumor development and invasiveness [62–64]. Ca^2+^ regulates the proliferation, migration, and lumen formation of endothelial cells through activating multiple important signaling pathway [65, 66]. Moreover, calcium signals interact with angiogenesis-related factors such as vascular endothelial growth factor (VEGF) and nitric oxide (NO), promoting the function of endothelial cells and the maturation of blood vessels [67]. Given the established role of calcium in angiogenesis and the known CCB activity of amlodipine, its potential effects on tumor vasculature need further explorations. Furthermore, while Hippo-YAP/TAZ pathway and EGFR/Akt data highlight the multimodal mechanisms of amlodipine, their crosstalk and cell-context dependencies require further elucidation. The chemo-sensitizing potential of amlodipine with TMZ in glioblastoma still needs future investigations. Hence, further translational studies are indispensable to fully realize the therapeutic promise of repurposing amlodipine for precision targeting therapy against glioblastoma.

## Conclusion

This study demonstrated that amlodipine exerts definite anti-GSC effects by inducing lysosome-dependent EGFR degradation via lipid raft-mediated endocytosis, thereby suppressing downstream pro-survival pathways of GSCs, which offers a promising therapeutic approach against development of glioblastoma.

## Materials and methods

### Cell lines and culture

Human glioma stem cell lines GSC11 and GSC23 (MD Anderson Cancer Center, USA) were cultured in Dulbecco’s modified Eagle’s medium (DMEM/F12) (Gibco, USA) with B27 supplement (1×, Gibco, USA), epidermal growth factor (EGF, 20 ng/mL) (Thermo Fisher Scientific, USA), and basic fibroblast growth factor (b-FGF, 20 ng/mL) (Thermo Fisher Scientific, USA). Human glioblastoma cell lines SNB19, T98G and LN229 (American Type Culture Collection, ATCC) were cultured in DMEM (Gibco, USA) supplemented with 10% fetal bovine serum (FBS, Gibco, USA). Normal human astrocytes (NHAs) (ScienCell, Carlsbad, CA, USA) were cultivated in DMEM medium supplemented with 10% FBS and 1% astrocyte growth supplement (ScienCell, USA). All cells were maintained in a cell incubator at 37 °C under humidified atmosphere containing 5% CO_2_. Additionally, these cell lines were validated to be free of mycoplasma contamination prior to the initiation of the experiment.

### Antibodies and reagents

The primary antibodies for cleaved PARP-1 (Cell Signaling Technology, CST, #5625, USA), caspase-3 (CST, #9662), cleaved caspase-3 (CST, #9661), caspase-9 (CST, #9502) cleaved caspase-9 (CST, #7237), CyclinD1 (CST, #2922), Bax (CST, #2772), Bcl-2 (CST, #3498), Akt (CST, #9272), p-Akt (Ser473) (CST, #9271), mTOR (CST, #2983), p-mTOR (CST, #2971), CD133 (CST, #64326), SOX2 (CST, #3579), EGFR (CST, #4267), p-EGFR (CST, #3777), Ubiquitin (CST, #20326), Na/K-ATPase (CST, #3010) and GAPDH (Proteintech, #60004-1-1, USA) were commercially available. Biochemical reagents including amlodipine (Selleck, S1905, USA), nifedipine (Selleck, S1808), nicardipine (Selleck, S5255), BAPTA-AM (Selleck, S7534), (s)-(-)-Bay-K-8644 (MCE, HY-15124, USA), CHX (MCE, HY-1320), SC79 (MCE, T2274), Baf A1 (MCE, HY-100558), MβCD (MCE, HY-101461) and MG132 (MCE, HY-13259) were purchased from the indicated suppliers.

### Cell viability analysis

Cell viability was evaluated with Cell Counting Kit-8 (CCK-8, Meilunbio, China). Briefly, 3 ×10^3^ cells/well were planted into 96-well plate and cultured with the indicated pharmaceuticals, then were incubated with CCK-8 solution (Meilunbio, China) at 37 °C for 2 h. Absorbance (OD value) was detected at wavelength of 450 nm with microplate reader (FilterMax F5, USA). The “log (inhibitor) vs normalized response-variable slope” method was applied to calculate the 50% inhibition concentration (IC50) of the indicated agent with GraphPad Prism 9.0 (GraphPad Software, USA).

### 5-Ethynyl-2’-deoxyuridine (EdU) assay

EdU Apollo567 in vitro Kit (RiboBio, C10310-1, China) was applied according to the manufacturer’s instructions. Cells were seeded into a 24-well plate and treated with the indicated pharmaceuticals. After incubation with 50 μM EdU for 2 h, cells were fixed in 4% paraformaldehyde and stained with Apollo Dye Solution. Cell nuclei were counterstained with 4’,6-diamidino-2-phenylindole (DAPI, Beyotime, China). EdU-positive cells were visualized under a fluorescence microscope (Leica, DMI8), images were captured, then analyzed with ImageJ (v1.8.0, NIH, USA).

### Cell cycle analysis

GSC23 and GSC11 cells were plated in 6-well plate at a density of 2 ×10^5^ cells/well, and treated with the indicated pharmaceuticals agents for 48 h. For cell cycle analysis, cells were harvested after digestion with StemPro™ Accutase™ (Thermo Fisher Scientific, USA), then washed with Phosphate-Buffered Saline (PBS, Thermo Fisher Scientific, USA), resuspended in 75% pre-chilled ethanol, and stored at 4 °C overnight. Next, cells were incubated with DNA-binding dye propidium iodide (PI, 50 µg/ml) and RNase (1.0 mg/ml) for 30 min at 37˚ °C in dark. After staining, cells were washed again with PBS, and red fluorescence was analyzed using a FACS Calibur flow cytometer (Becton Dickinson, San Diego, CA, USA). A peak fluorescence gating strategy was applied to discriminate single cells from aggregates during analysis.

### Three-dimensional (3D) spheroid invasion assay

Matrigel (Corning, USA), micropipette tips, and 96-well plate were pre-chilled overnight at 4 °C. GSC23 and GSC11 cells were cultured in medium favored for stem cell growth. Tumor spheres of GSCs about of 30–50 μm in diameter were transferred to 96-well plate. Matrigel was mixed with an equal amount of DMEM/F12 culture medium on ice, and 200 µl mixture were added into each well of 96-well plate. The indicated pharmaceuticals were diluted in DMEM/F12 medium. Then the plate was placed in incubator and continued cultivation for 48 h. Images were captured every 24 h using an inverted microscope. The invasion distance and area of GSC spheroids were quantified using ImageJ software.

### Tumor sphere formation assay

Cells were seeded at a density of 500 cells/well in 96-well plate and incubated at 37 °C in a humidified atmosphere with 5% CO_2_.The following day, the indicated compounds were added to each well at final concentrations ranging from 0 to 25 µM, (with 5 µM intervals). After 7 days of culture, the number and size of GSC spheres were recorded under a microscope and quantified using ImageJ software.

### Annexin V-fluorescein isothiocyanate/propidium iodide (Annexin V-FITC/PI) assay

GSC11 and GSC23 cells were treated with indicated pharmaceuticals for 48 h. Cells were then harvested and subjected to Annexin V-FITC/PI Apoptosis Detection kit (MULTI Sciences, China), according to the manufacturer’s instructions. Analysis was performed with a flow cytometer (Becton Dickinson, San Diego, CA, USA). The percentage of cell apoptosis was analyzed with FlowJo Version 10 software.

### Terminal deoxynucleotidyl transferase-mediated dUTP Nick-End Labeling (TUNEL) staining

Terminal deoxynucleotidyl TUNEL apoptosis detection was performed to label the 3′-end of fragmented DNA of apoptotic cells (One Step TUNEL Apoptosis Assay Kit, Elabscience, China), following the manufacturer’s instructions. Briefly, prepared cells or tissue sections were permeabilized with 0.2% Triton X-100, and labeled with TUNEL working solution in dark. Nuclei were stained with DAPI. TUNEL-positive cells were visualized under a fluorescence microscope, images were captured and analyzed with ImageJ.

### Western blot

Collected cells or minced fresh tissues were lysed with standard lysis buffer containing protease and phosphatase inhibitors (Sigma-Aldrich, USA). Then lysate was centrifuged to collect supernatant. Protein concentration was determined with Bicinchoninic Acid assay (BCA, Thermo Fisher Scientific, USA). A total of 20–30 μg of protein sample was subjected to 10% Sodium Dodecyl Sulfate - Polyacrylamide Gel Electrophoresis (SDS-PAGE) gel, then transferred onto 0.22-mm polyvinylidene difluoride (PVDF) membrane (NEN Research Products, USA). The membrane was blocked in 5% non-fat milk for 2 h at room temperature. The primary antibody was applied at 4 °C overnight. After incubation with the primary antibody, the membrane was washed three times with PBS with 0.1% Tween 20 for 10 min, then incubated with the appropriate HRP-conjugated second antibodies. The binding was visualized using Chemiluminescence Reagent Plus (PerkinElmer, USA). Protein signals were captured on X-ray films (Kodak BioMax MR). Densitometric analysis was performed using ImageJ software, and original films are provided in the Supplementary Materials.

### Co-immunoprecipitation (Co-IP)

Cells were lysed in immunoprecipitation buffer (Thermo Fisher, USA) with protease inhibitor cocktail. The cell lysate was incubated with monoclonal anti-EGFR antibody at 4 °C overnight, followed by incubation with avidin-conjugated agarose beads at room temperature for 2 h. Immune complexes were washed five times with immunoprecipitation buffer, then eluted through boiling in 2× loading buffer for 5 min. Protein levels of co-immunoprecipitated proteins were then assessed by Western blot analysis.

### Quantitative reverse transcription - polymerase chain reaction (qRT-PCR)

Collected cells were lysed with TRIzol (Sigma-Aldrich, USA) to extract total RNA. Complementary DNA (cDNA) synthesis was performed with reverse transcription cDNA Kit (Thermo Fisher, USA), according to the manufacturer’s instructions. SYBR PrimeScript RT-PCR Kit (Novoprotein, China) was applied to detect PCR amplification products.

The qPCR data about relative gene expression levels were analyzed using the 2^−ΔΔCt^ method, with GAPDH used as the internal control. The primers used in this study were designed and synthesized by Sangon Biotech (Shanghai, China). The following primers were used to quantify mRNA level of target genes: GAPDH (forward, 5′-GGAGCGAGATCCCTCCAAAAT-3′; reverse, 5′-GGCTGTTGTCATACTTCTCATGG-3′), CD133 (forward, 5′-AGTCGGAAACTGGCAGATAGC-3′; reverse, 5′-GGTAGTGTTGTACTGGGCCAAT-3′), SOX2 (forward, 5′-GCCGAGTGGAAACTTTTGTCG-3′; reverse, 5′-GGCAGCGTGTACTTATCCTTCT-3′). EGFR (forward, 5′-CCTGGTCTGGAAGTACGCAG-3′; reverse, 5′-CGATGGACGGGATCTTAGGC-3′).

### Immunofluorescence (IF) and immunohistochemistry staining (IHC)

For IF staining, prepared cells were fixed in 4% paraformaldehyde fix solution (PFA) for 30 min, washed with PBS for 3 times, and permeabilized in PBS containing 0.5% Triton X-100 (Solarbio, T8200) for 20 min. Cells were blocked with 5% goat serum (Solarbio, SL038) at room temperature for 1 h, and incubated with the indicated primary antibody at 4 °C overnight, then incubating with the corresponding second fluorescence-labeled antibody at room temperature for 1 h. The nuclei were counterstained with DAPI (Invitrogen). Images were acquired via laser confocal microscope (Leica, Germany) and processed utilizing ImageJ software.

For IHC staining, the whole brains of tumor-bearing mice were harvested under general anesthesia, fixed with 4% PFA, and embedded in paraffin. Then, 5μm-slice tissue sections were prepared continuously with microtome (Leica, Germany), followed subsequentially with deparaffinization, dehydration, and incubation in heat-mediated antigen retrieval. Endogenous catalase was eliminated with 3% H_2_O_2_–methanol, and tissue slices were incubated with the indicated primary antibody at 4 °C overnight. After washing with PBS, tissue sections were incubated with the biotinylated second antibody at room temperature for 1 h, then were incubated with peroxidase solution for 30 min. The sections were stained with 3,3’-Diaminobenzidine (DAB) reagent, counterstained with hematoxylin, observed under optical microscopic view, and images were captured and analyzed with ImageJ.

### Molecular docking analysis

The X-ray crystal structures of EGFR (PDB: 1NQL) were retrieved from the Protein Data Bank (https://www.rcsb.org/). The protonation state of all the compounds was set at pH 7.4, and their 3D structures were generated using Open Babel. AutoDock Tools (ADT3) were applied to prepare and parametrize the receptor protein and ligands. The docking grid were generated by AutoGrid, and AutoDock Vina (v1.2.0) was applied for docking simulation. The optimal pose was selected to analyze the interactions. Finally, the figures of protein-ligand interactions were generated by PyMOL.

### Intracranial xenograft model

Female athymic BALB/c nude mice (aged 4 weeks, weight 15–20 g, from Shanghai laboratory animal Center, Shanghai, China) were housed in specific pathogen-free (SPF) environment acclimatized to their surroundings with water and food provided ad libitum for 1 week.

For establishing an intracranial tumor-bearing model, GSC23 cells were transduced with firefly luciferase, and then GSC23-Luc cells (1 ×10^6^) were suspended in 10 μl PBS and injected slowly into the right caudate nucleus with stereotactic techniques. One week after transplantation of GSCs, mice bearing GSC-derived intracranial xenografts were randomly divided into 3 groups (*n* = 5/group), including the control group (vehicle), amlodipine group (10 mg/kg/day, intraperitoneal injection for 3 consecutive weeks), and amlodipine plus SC79 group (intraperitoneal injection of amlodipine and SC79 (10 mg/kg/day) for 3 weeks). Based on scientific goals and animal ethics, we believe that this sample size suffices for experiment and ensures reliable data for research. Meanwhile, in this study, blinding was not adopted because the treatments were visually distinguishable.

Bioluminescent imaging was performed to monitor tumor growth at indicated days. Mice were treated with d-luciferin (0.15 mg/g; GoldBio, LUCK10G) by intraperitoneal injection and anesthetized with isoflurane before imaging. The luciferase fluorescent signals were captured under an IVIS imaging system (IVIS Lumina XP, PerkinElmer, USA). Overall survival was recorded for each group. All animal experiments were approved by the Institutional Animal Care and Use Committee of Soochow University (Approval Number: SUDA 20210708A03).

### Statistical analysis

All statistical analyses were conducted with GraphPad Prism 9.0 (GraphPad Software, USA). Results are presented as mean ± standard deviation (SD) from at least three independent experiments. The normality of data distribution was analyzed by Shapiro–Wilk test. Student’s *t* test was performed to analyze the statistical difference between two groups, and analysis of variance (ANOVA) was applied to evaluate the differences between multiple groups. The Levene test was employed to assess the homogeneity of variances across the groups. For analysis of animal survival, the Kaplan–Meier curve method with the log-rank test for comparison was applied. The *p*-value < 0.05 was considered statistically significant (*p < 0.05, **p < 0.01, ***p < 0.001, ****p < 0.0001). *p*-Value > 0.05 was considered not significant (NS).

## Supplementary information

Supplement figures and legends

Original western blots

## Data Availability

The data generated in this study are available upon request from the corresponding author.
