# Supplementary material for: Amlodipine exerts inhibitory effects against glioma stem cells through degrading EGFR and down-regulating its downstream pro-survival pathways
Source: Cell Death Discov. 2025 Oct 27;11:492. doi: 10.1038/s41420-025-02784-3 (PMC12559749; doi:10.1038/s41420-025-02784-3)
Supplement: Supplementary file 1 — Supplement figures and legends [file 41420_2025_2784_MOESM1_ESM.docx]

**Supplement Figure**

**Figure S1**

**
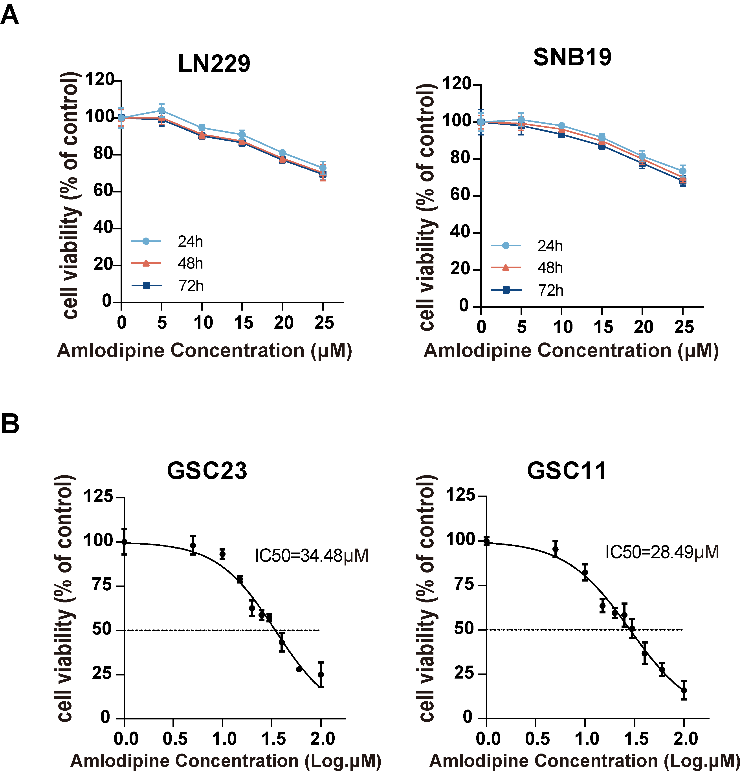
**

**Fig. S1 Amlodipine had exact inhibitory effect on cell viability of GSCs.**

(**A**) Incubation of SNB19 and LN229 cells with indicated concentrations of amlodipine (varying from 0 to 25 μM, with 5 μM interval) for 24, 48 and 72h, respectively. The effect on cell viability of LN229 cells was determined by CCK8 assay**.** (**B**) Incubation of GSC23 and GSC11 cells with various concentrations (1, 5, 10, 15, 20, 25, 30, 40, 60 and 100 μM) of amlodipine for 48h. The “log (inhibitor) versus normalized slope of response variable” method was applied to calculate the 50% inhibition concentration (IC50) of the amlodipine.

**Figure S2**

**
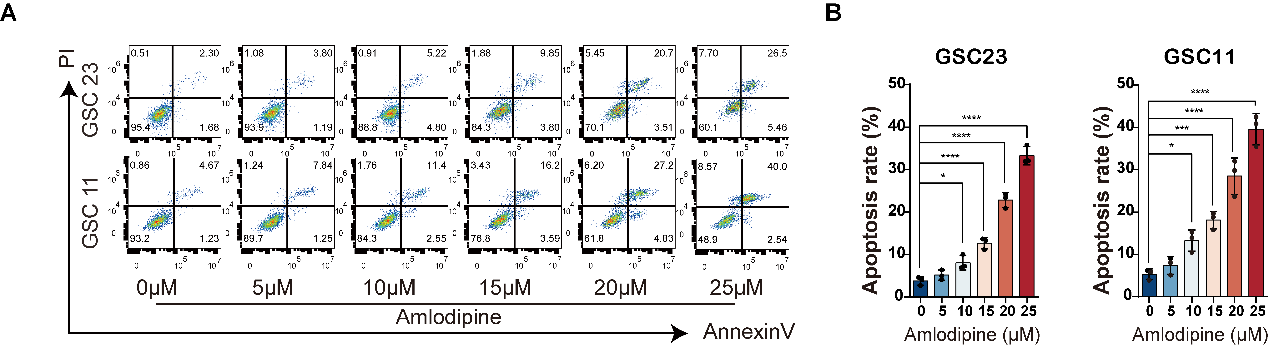
**

**Fig. S2 Amlodipine promoted apoptosis of GSCs**

(**A**) GSC23 and GSC11 cells were harvested after 48 h treatment with indicated concentrations of amlodipine (varying from 0 to 25 μM, with 5 μM interval) for 48h, and apoptotic cells were analyzed by Annexin VFITC–PI dual staining flow cytometry. (**B**) Histograms showing mean percentage of apoptotic cells.

**Figure S3**


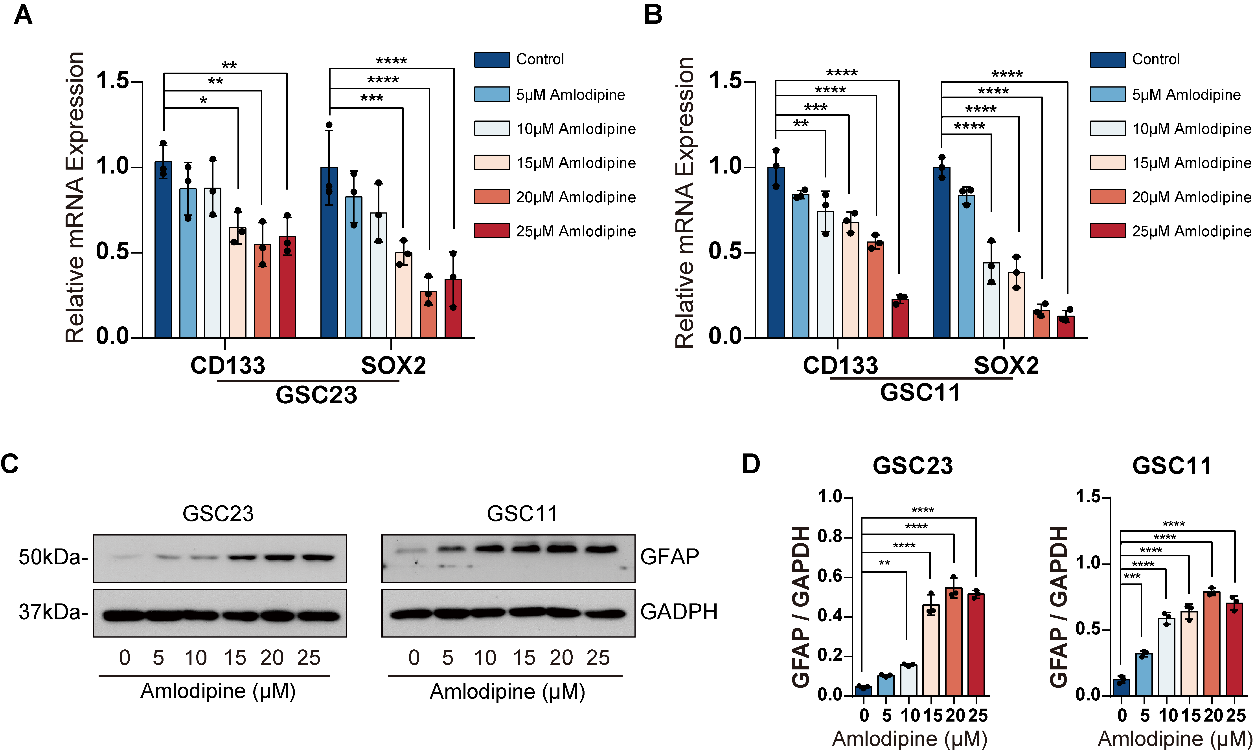


**Fig. S3 Amlodipine inhibited stemness of GSCs.**

(**A-B**) qRT-PCR analysis of tumor stem cell markers CD133 and SOX2 expression in GSC23 and CSC11 cells after treated with indicated concentrations of amlodipine (varying from 0 to 25 μM, with 5 μM interval) for 48h, qRT-PCR was performed with specific primers. (**C-D**) Western blot and quantitative analysis of the expression level of GFAP in GSC23 and CSC11 cells after treatment with indicated concentrations of amlodipine (varying from 0 to 25 μM, with 5 μM interval) for 48h. GAPDH was applied as the loading control.

**Figure S4**

**
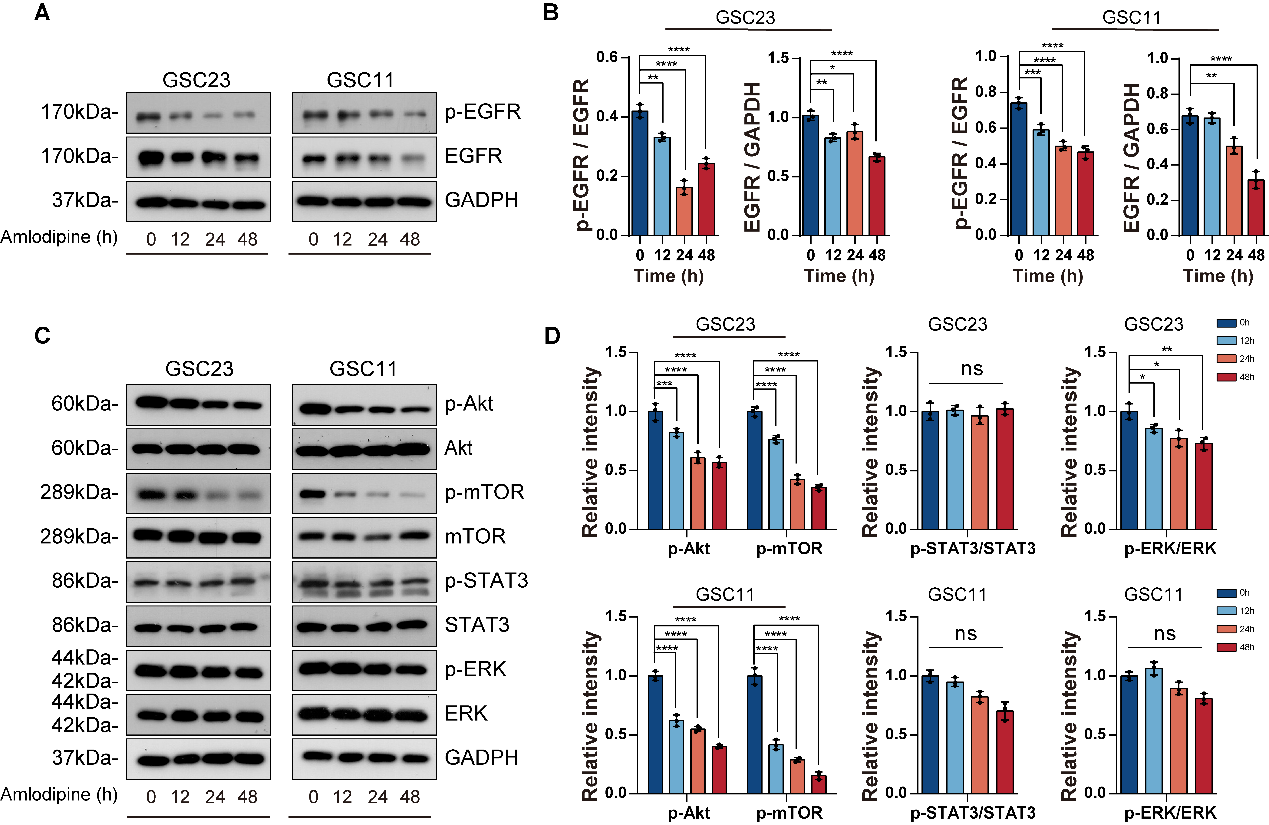
**

**Fig. S4 Amlodipine suppressed EGFR and its downstream pro-survival pathways of GSCs.**

(**A**) Western blot to assay the total and phosphorylated EGFR level of GSC23 and GSC11 cells after treated with indicated amlodipine (15μM) for 0, 12, 24 and 48h, respectively. (**F**) Quantitative analysis of total EGFR level, and p-EGFR/EGFR ratio.

(**G**) Western blot to detect expression level of Akt, p-Akt, mTOR, p-mTOR, ERK, p-ERK, STAT3 and p-STAT of GSC23 and GSC11 cells after addition of indicated amlodipine (15μM) for 0, 12, 24 and 48h, respectively. (**H**) Quantitative analysis of the ratio of p-AKT/AKT, p-mTOR/mTOR, p-STAT3/STAT3, and p-ERK/ERK expression level.
