# Supplementary material for: Amlodipine exerts inhibitory effects against glioma stem cells through degrading EGFR and down-regulating its downstream pro-survival pathways
Source: Cell Death Discov. 2025 Oct 27;11:492. doi: 10.1038/s41420-025-02784-3 (PMC12559749; doi:10.1038/s41420-025-02784-3)
Supplement: Supplementary file 2 — Original western blots [file 41420_2025_2784_MOESM2_ESM.docx]

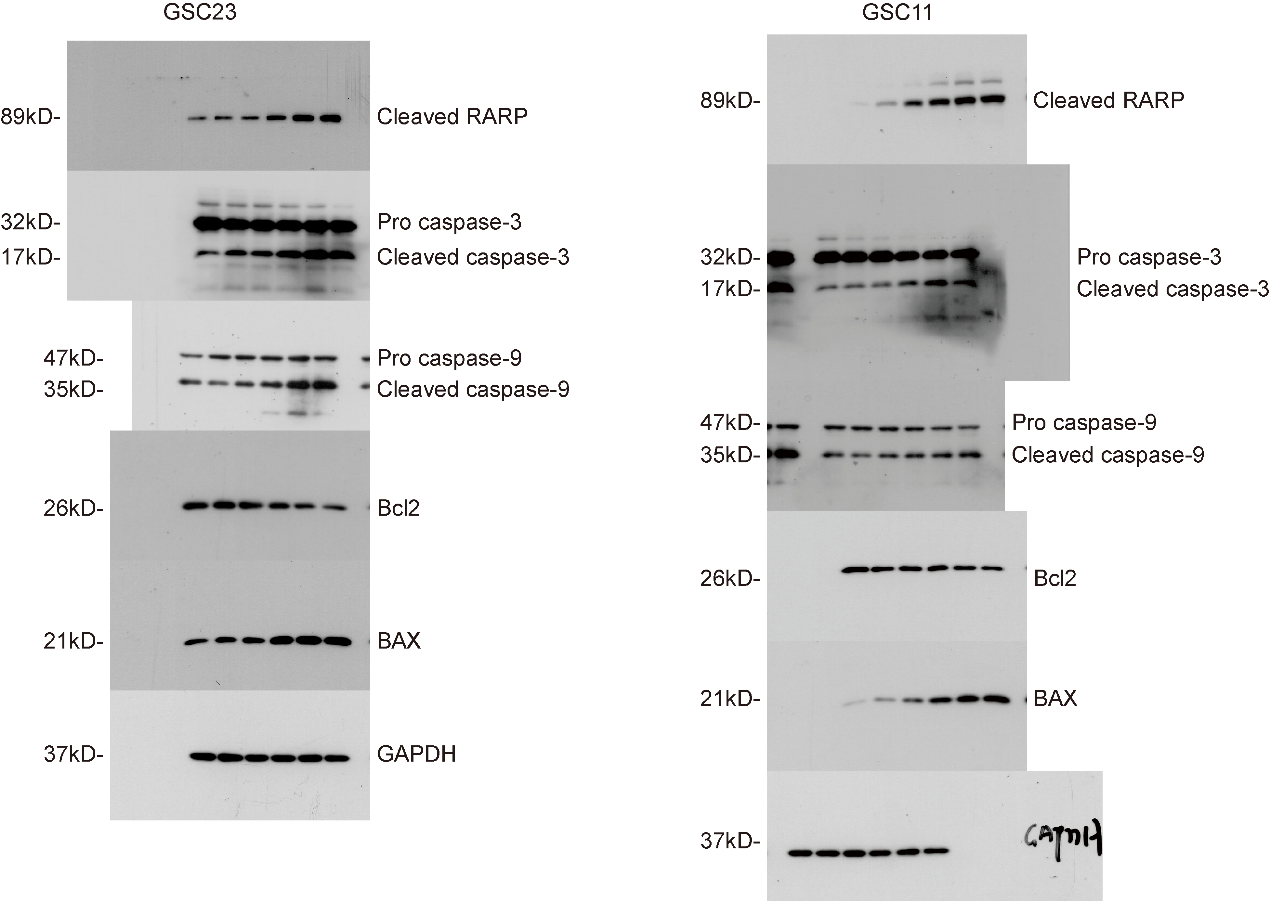


The complete set of original autoradiograph films corresponding to Figure 2C.


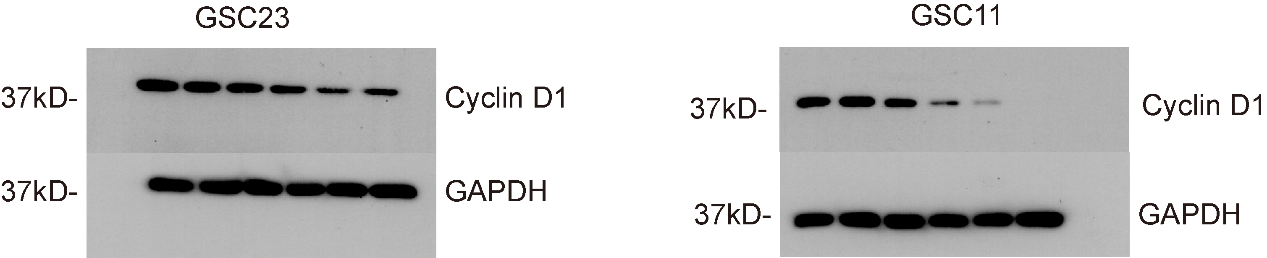


The complete set of original autoradiograph films corresponding to Figure 2G.


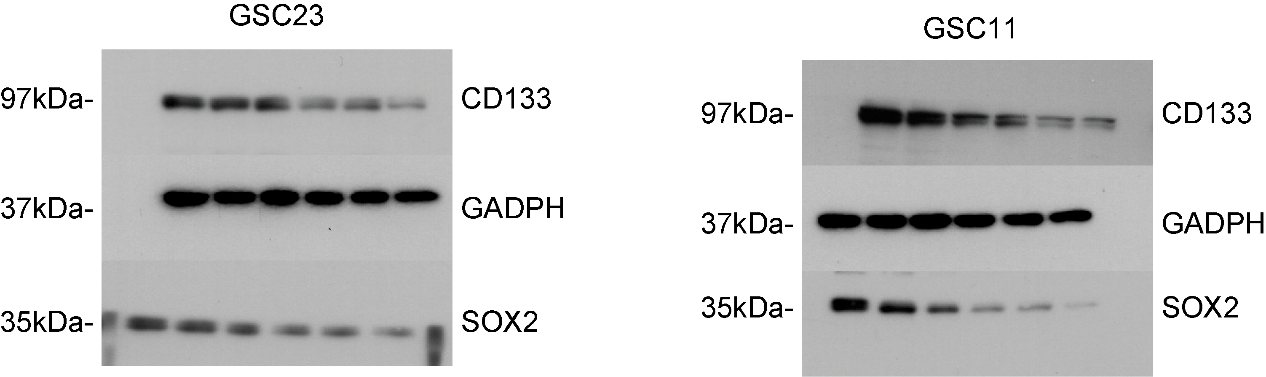


The complete set of original autoradiograph films corresponding to Figure 3D.


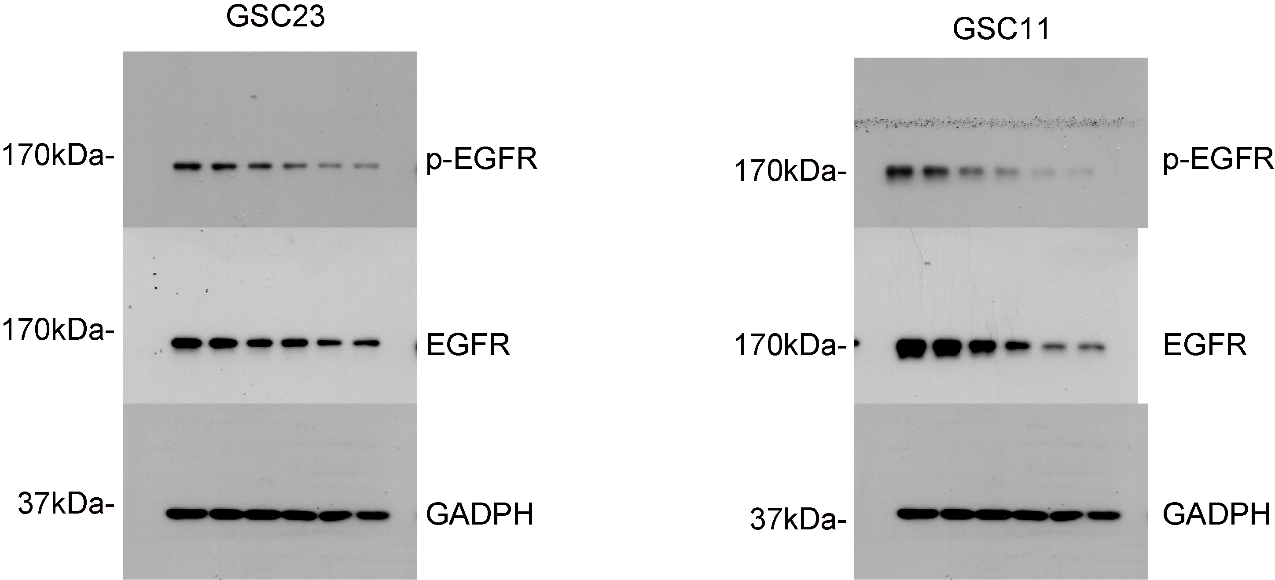


The complete set of original autoradiograph films corresponding to Figure 4E.


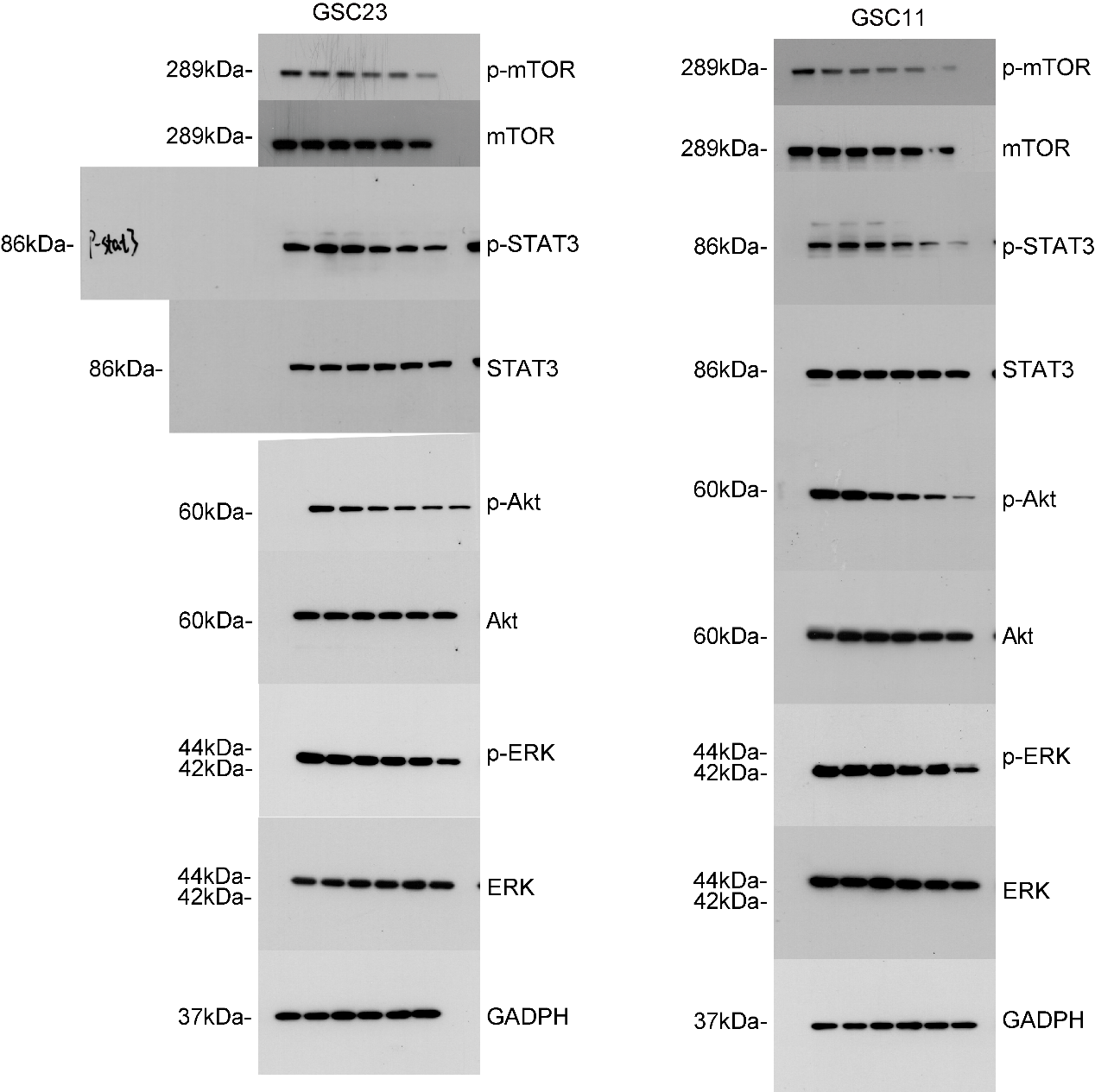


The complete set of original autoradiograph films corresponding to Figure 4G.


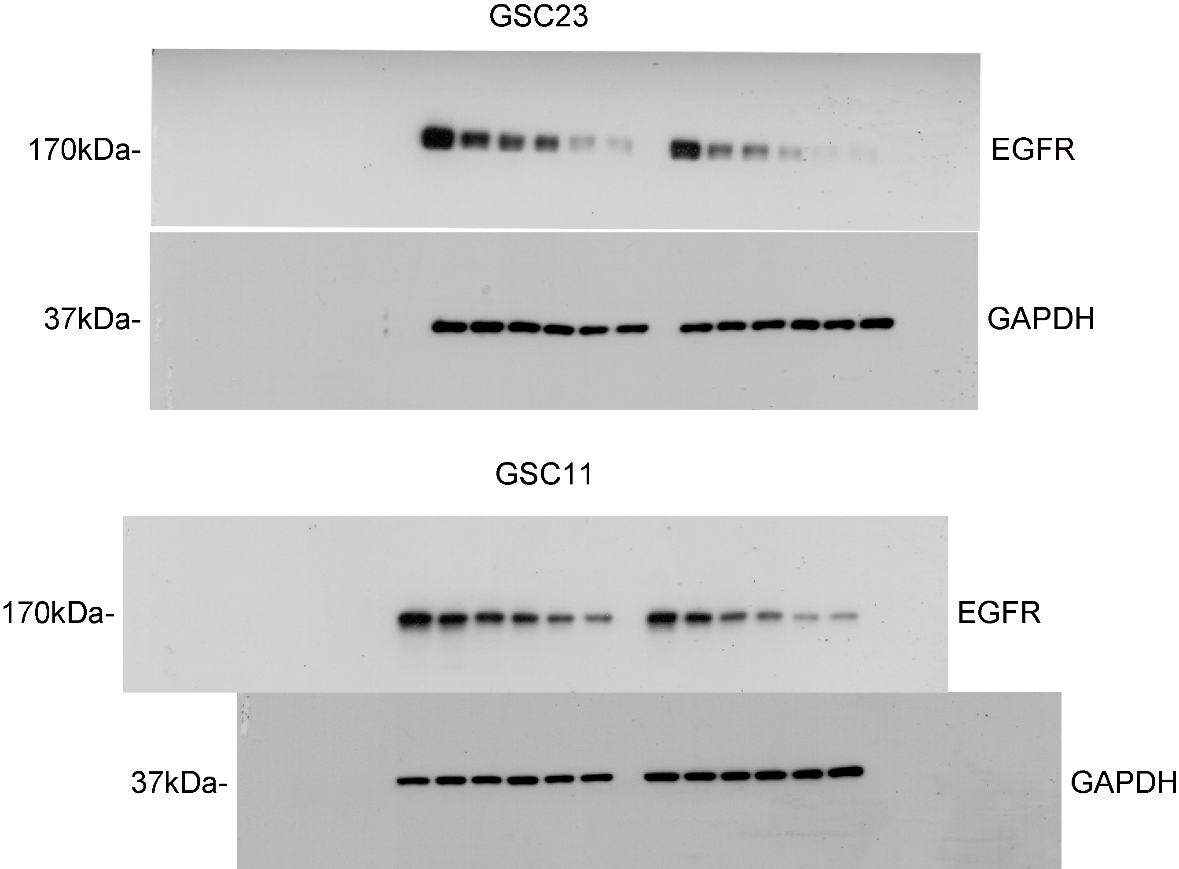


The complete set of original autoradiograph films corresponding to Figure 5B.


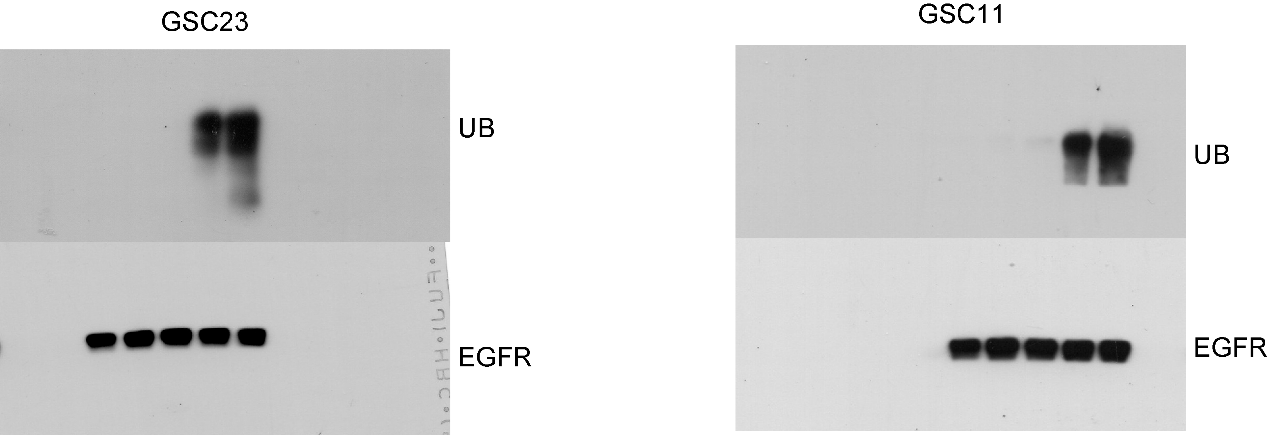


The complete set of original autoradiograph films corresponding to Figure 5C.


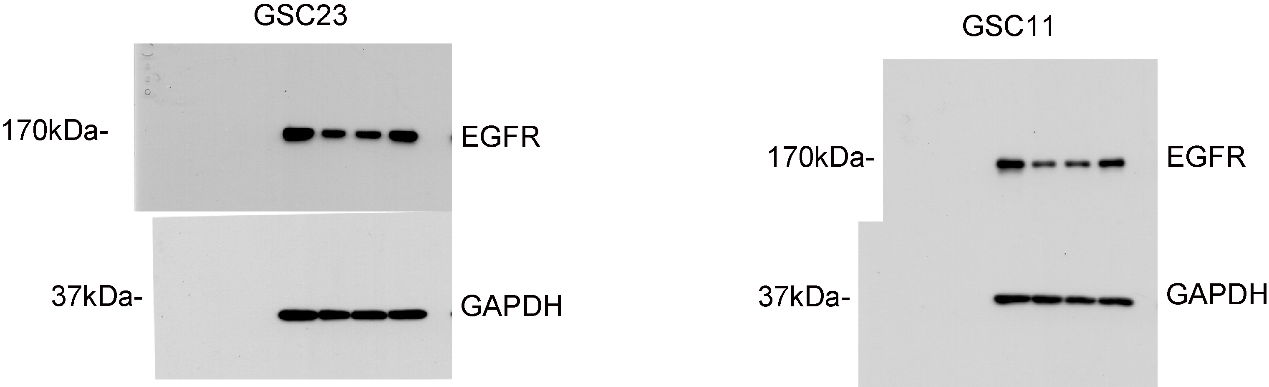


The complete set of original autoradiograph films corresponding to Figure 5D


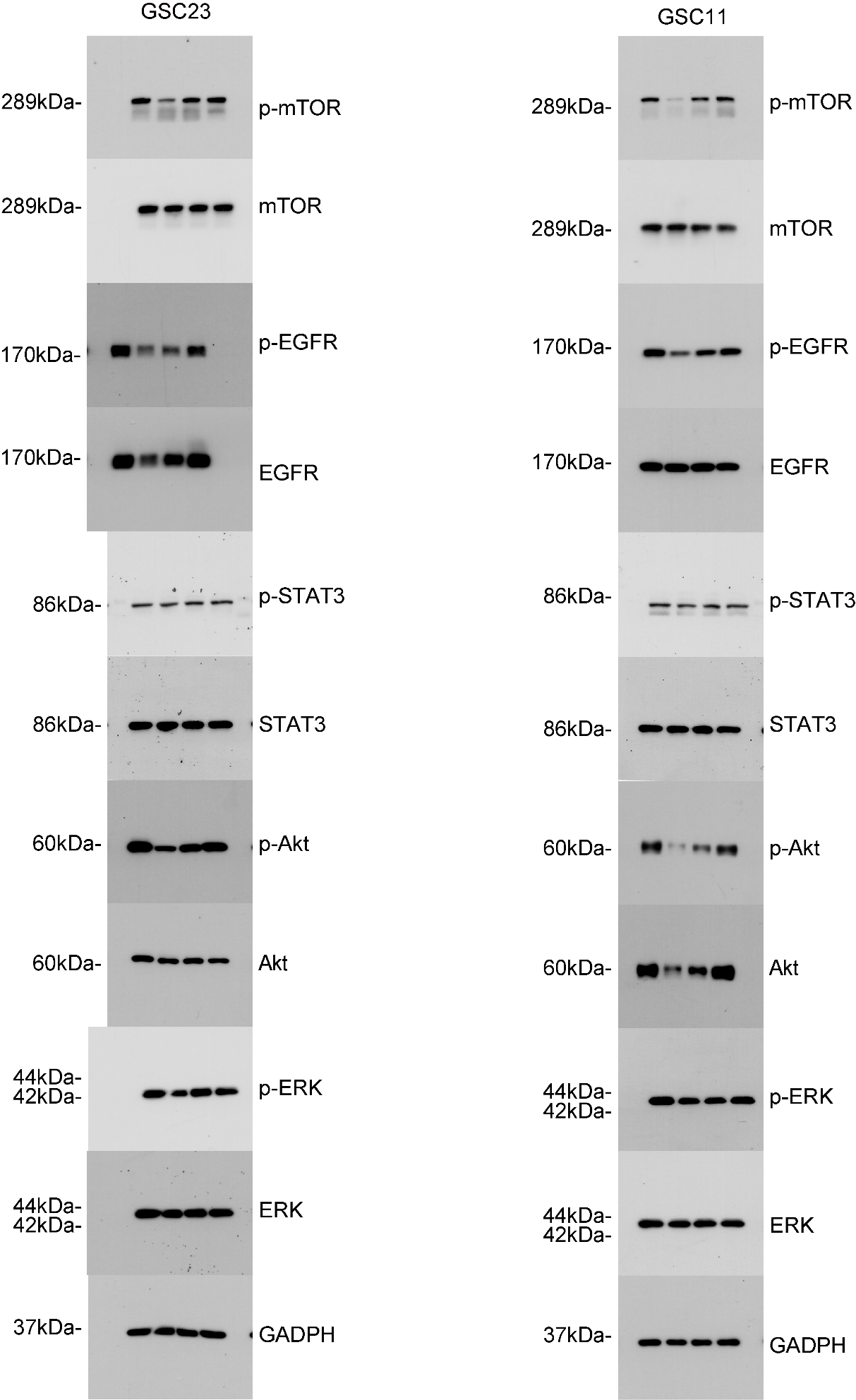


The complete set of original autoradiograph films corresponding to Figure 5F


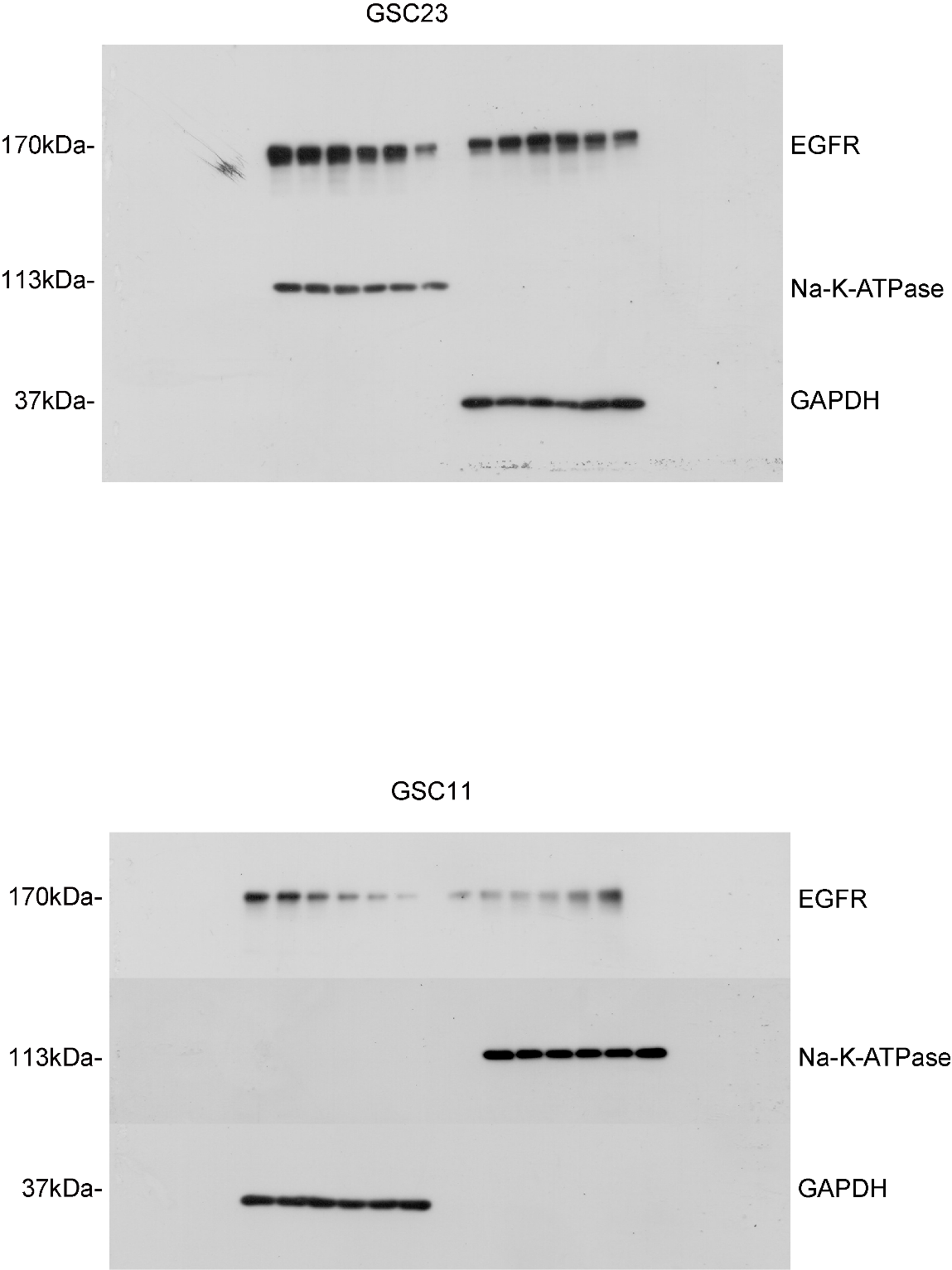


The complete set of original autoradiograph films corresponding to Figure 6B.


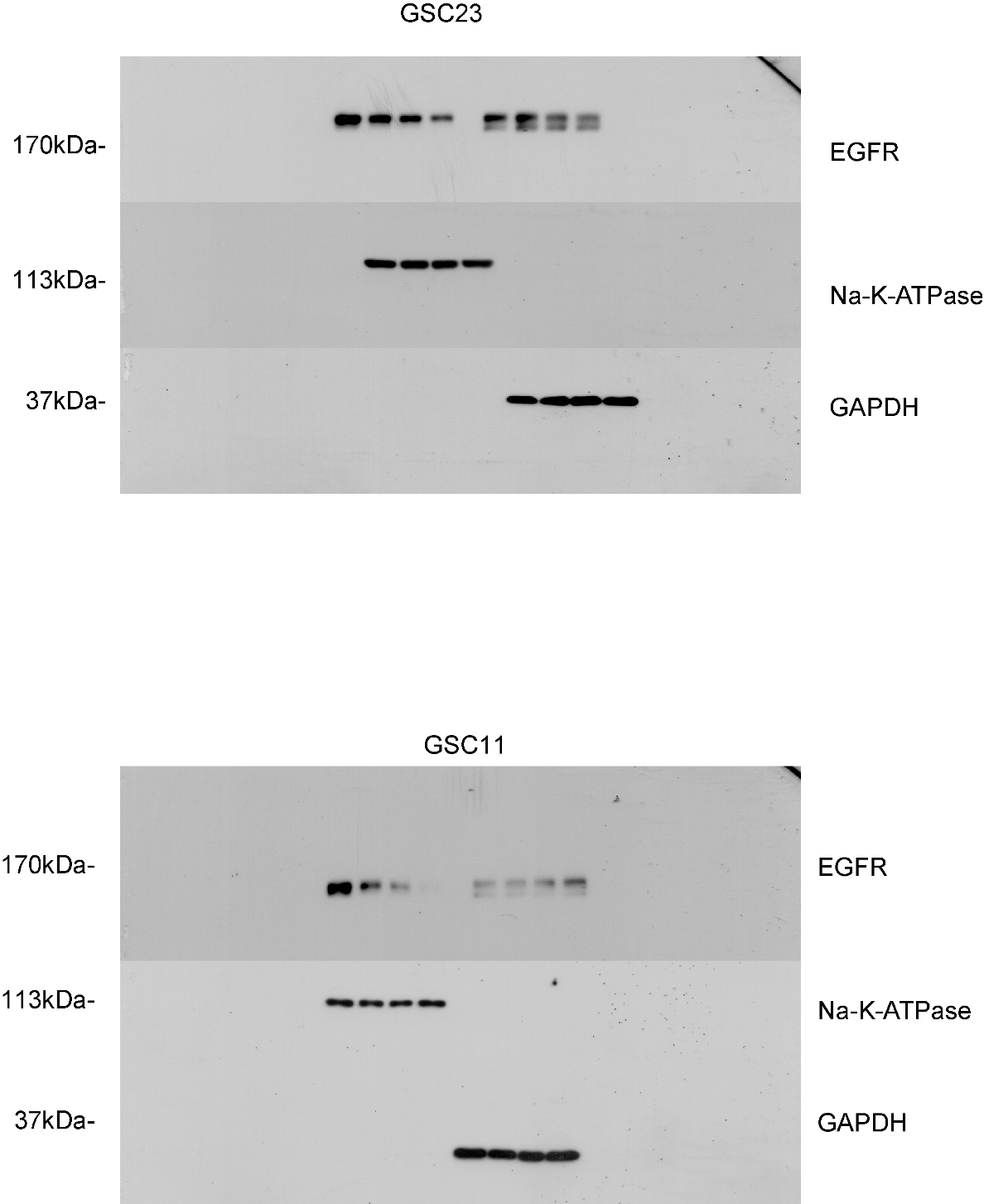


The complete set of original autoradiograph films corresponding to Figure 6C.


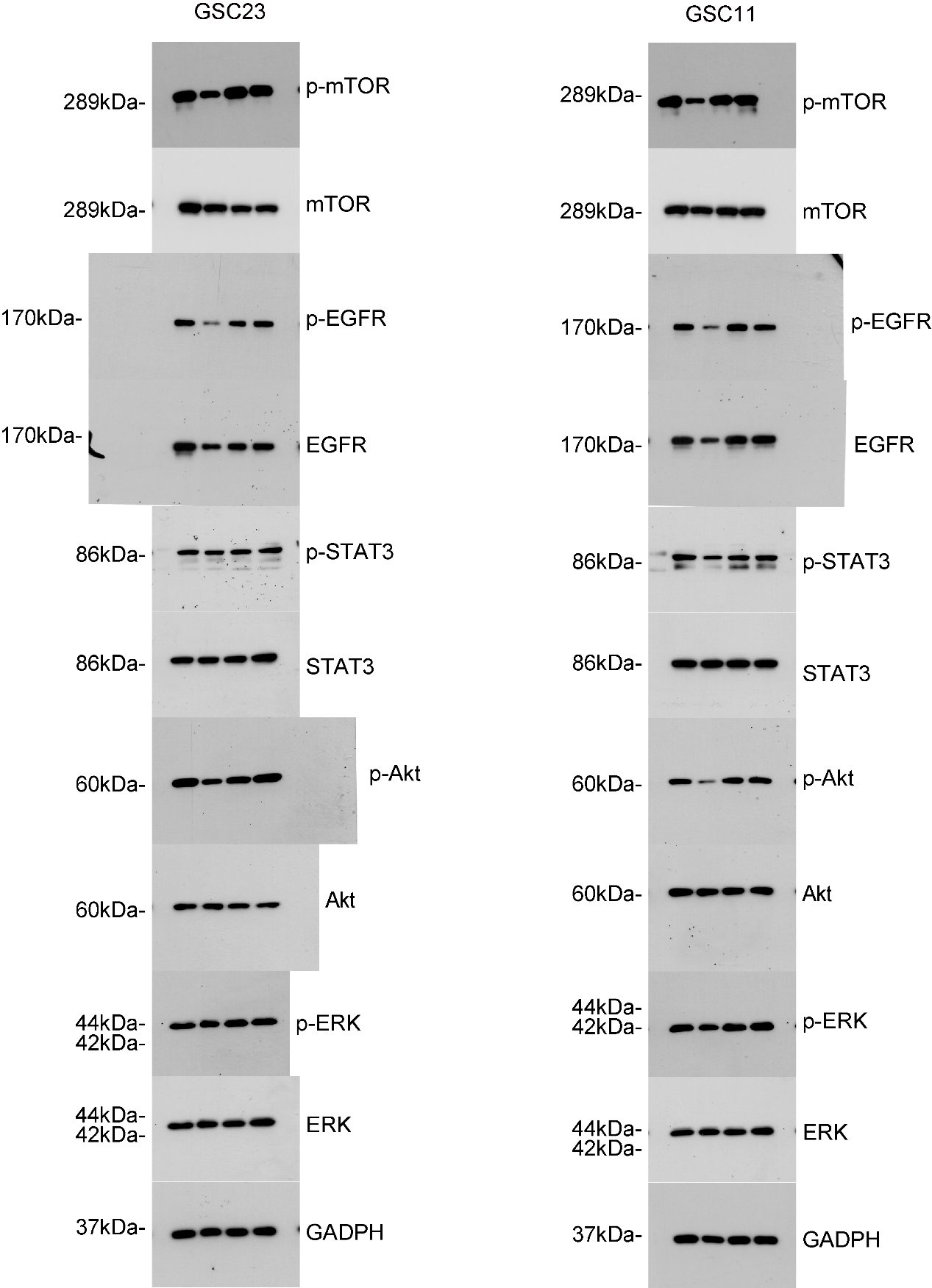


The complete set of original autoradiograph films corresponding to Figure 6E.


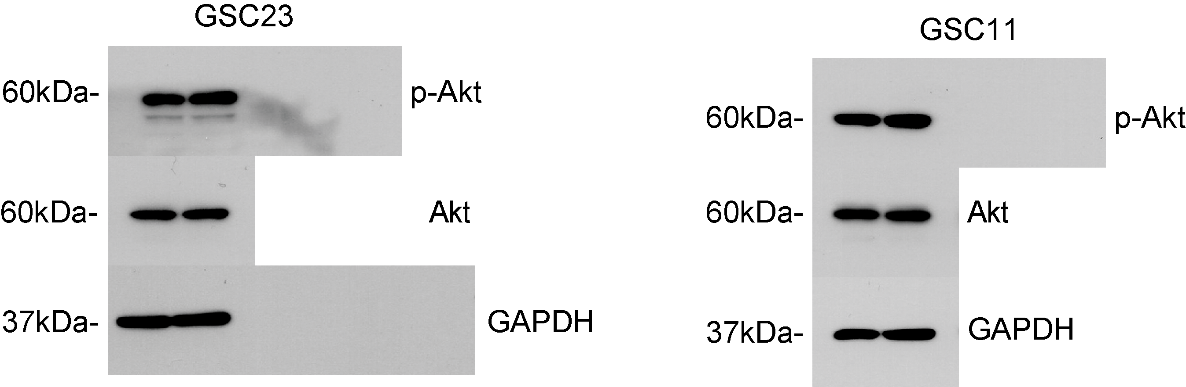


The complete set of original autoradiograph films corresponding to Figure 7A.


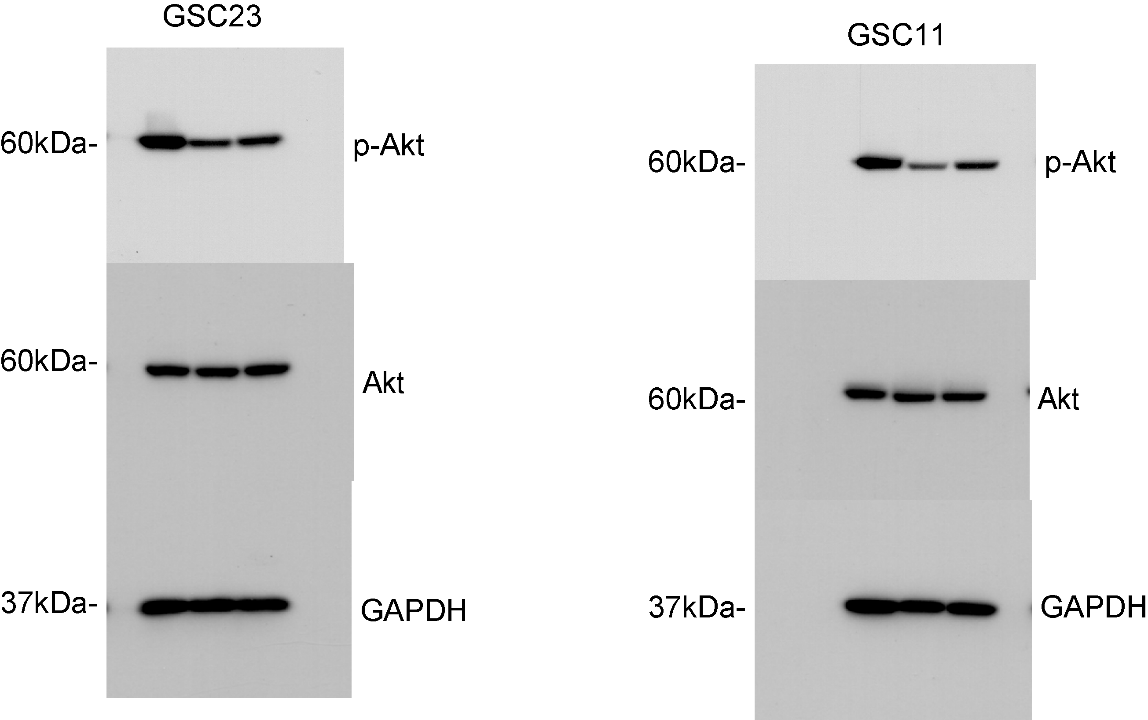


The complete set of original autoradiograph films corresponding to Figure 7B.


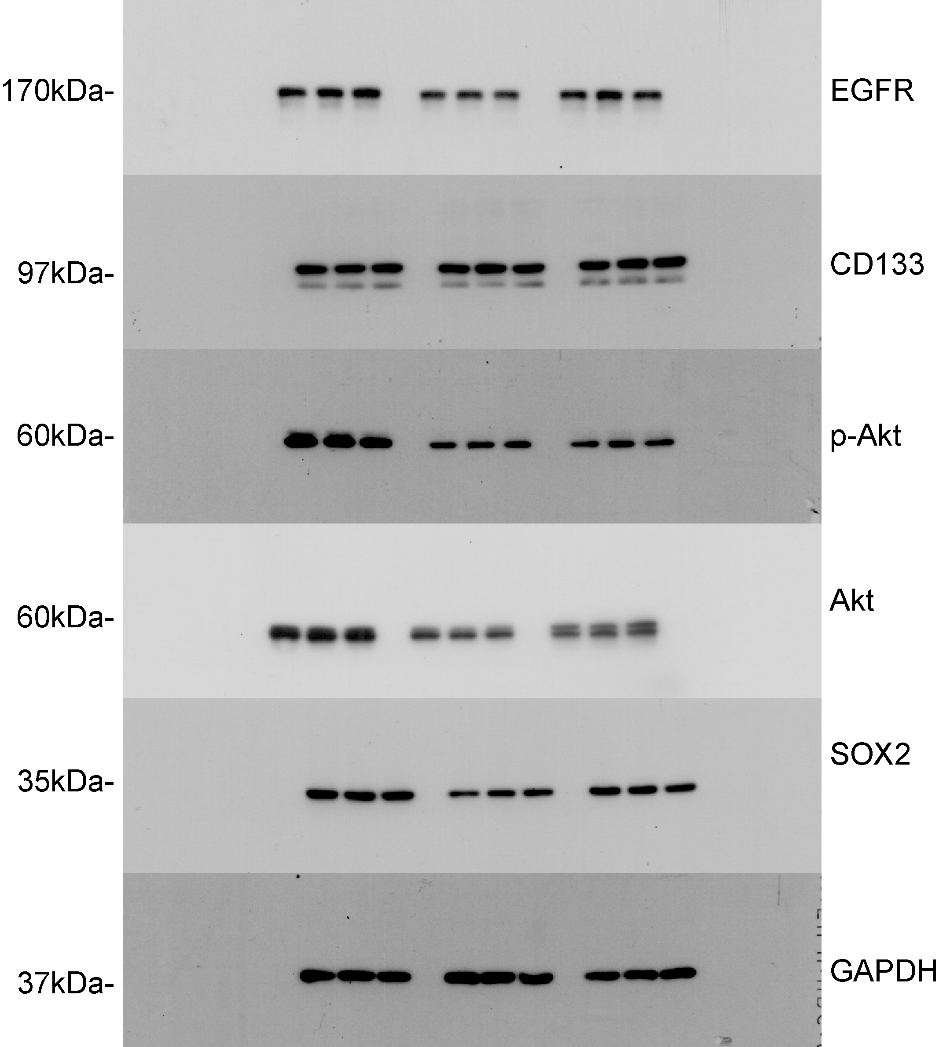


The complete set of original autoradiograph films corresponding to Figure 8I.


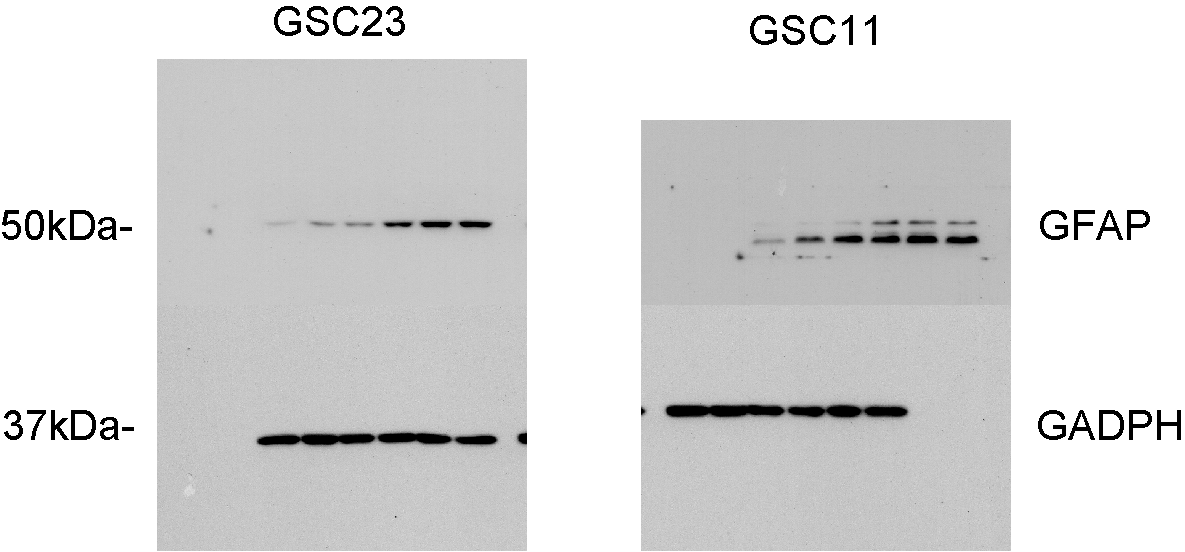


The complete set of original autoradiograph films corresponding to Figure S3C.


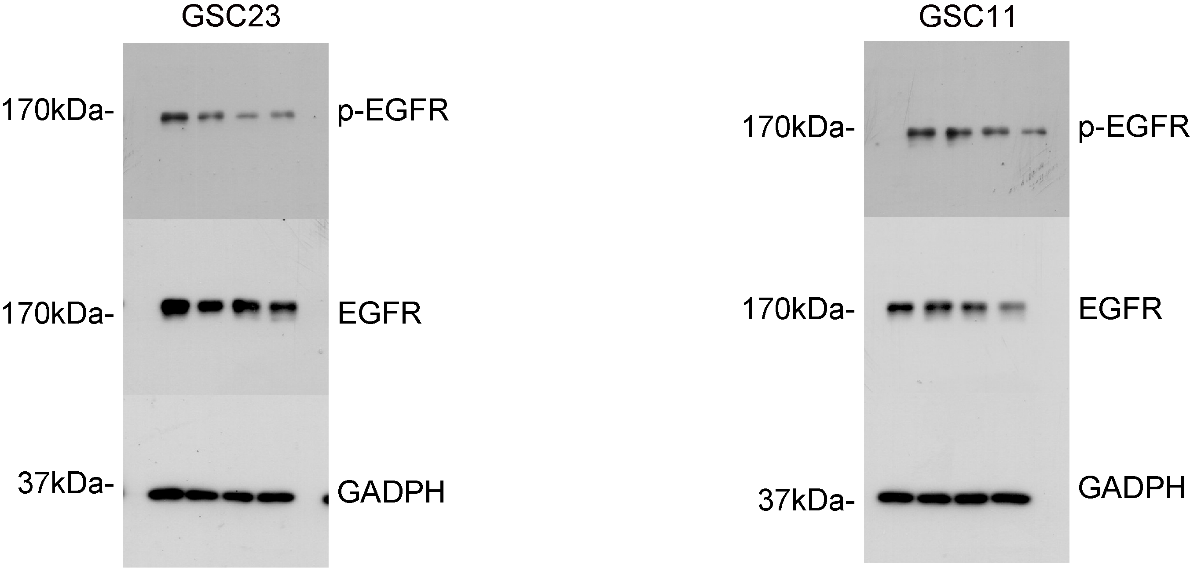


The complete set of original autoradiograph films corresponding to Figure S4A.


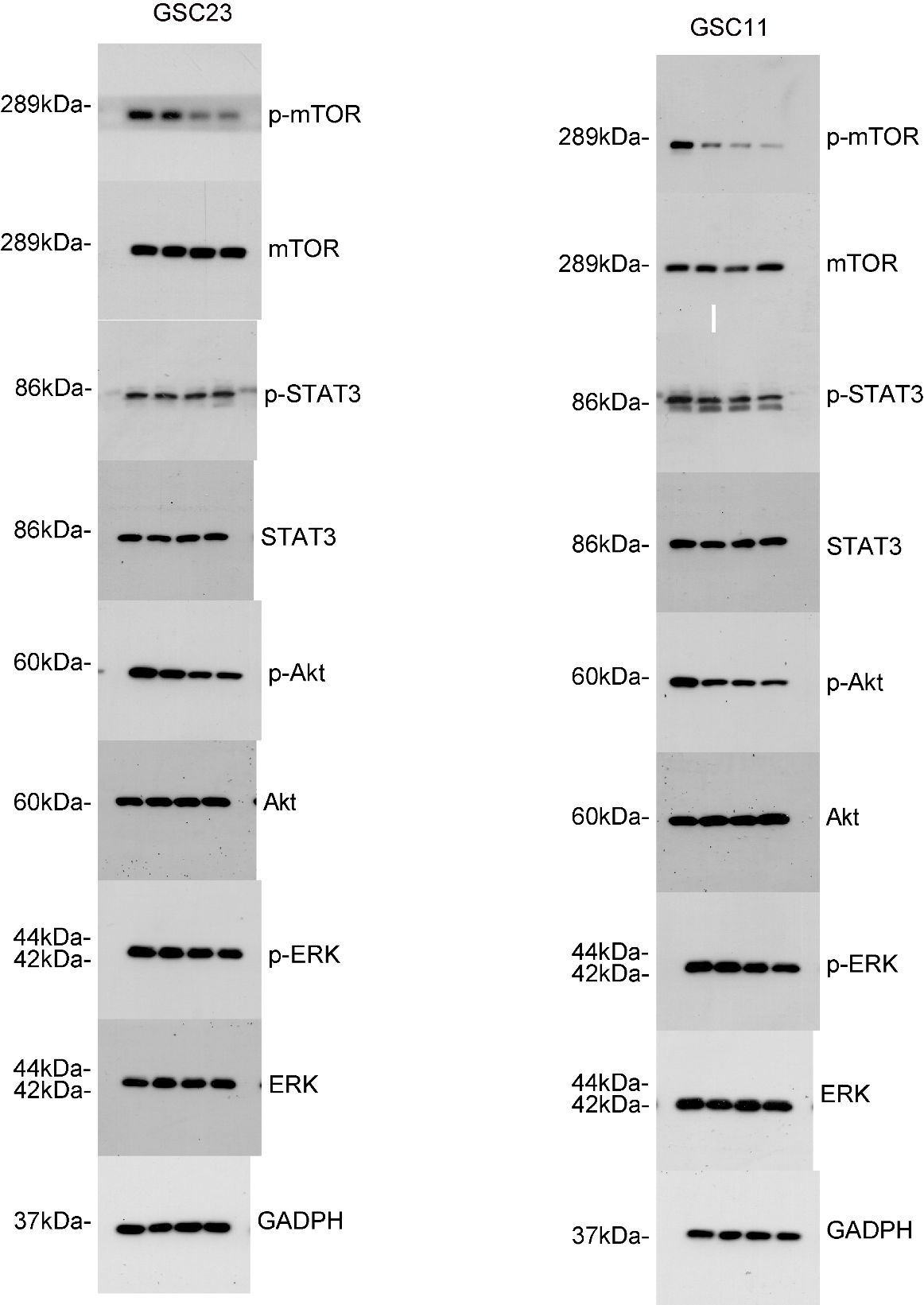


The complete set of original autoradiograph films corresponding to Figure S4C.
